# Efficacy and safety of first-line immunotherapy plus chemotherapy in treating patients with extensive-stage small cell lung cancer: a Bayesian network meta-analysis

**DOI:** 10.3389/fimmu.2023.1197044

**Published:** 2023-06-26

**Authors:** Tianming Zhang, Wenjun Li, Danbei Diwu, Lijun Chen, Xi Chen, Hong Wang

**Affiliations:** ^1^ Department of Respiratory Medicine, Lanzhou University Second Hospital, Lanzhou, Gansu, China; ^2^ School of Health, Brooks College (Sunnyvale), Sunnyvale, CA, United States; ^3^ Department of Epidemiology and Statistics, School of Public Health, Medical College, Zhejiang University, Hangzhou, Zhejiang, China

**Keywords:** extensive-stage small cell lung cancer, immunotherapy, network meta-analysis, efficacy, safety

## Abstract

**Background:**

Despite numerous immunotherapy and chemotherapy regimens available for patients with extensive-stage small cell lung cancer (ES-SCLC), it remains unclear which regimen is the most effective and safest; relative studies comparing such regimens are scarce.

**Objective:**

The aim of this study was to investigate the efficacy and safety of first-line immunotherapy combinations with chemotherapy for patients with extensive-stage small cell lung cancer. In addition, for the first time, comparisons among the first-line systemic regimens on OS and PFS in ES-SCLC by each time node were made.

**Methods:**

Databases including PubMed, Embase, Cochrane Library, Scopus, Google Scholars, and ClinicalTrials.gov, and major international conferences were searched for randomized controlled trials (RCTs) regarding comparing immunotherapy combinations with chemotherapy as first-line treatments for patients with advanced ES-SCLC from inception to 1 November. Hazard ratios (HRs) and odds ratios (ORs) were generated for dichotomous variants by RStudio 4.2.1. The outcomes comprised overall survival (OS), progression-free survival (PFS), objective response rate (ORR), and adverse events of grade 3 or higher (Grade ≥ 3 AEs).

**Results:**

Eventually, a total of nine RCTs reporting 4,352 individuals with nine regimens were enrolled. The regimens were ipilimumabnu (Ipi), atezolizumab (Atez), durvalumab plus tremelimumab (Durv-Trem), durvalumab (Durv), pembrolizumab (Pemb), adebrelimab (Adeb), serplulimab (Serp), atezolizumab plus tiragolumab (Atez-Tira), and nivolumab (Nivo). With regard to OS, serplulimab (HR = 0.63, 95% CI: 0.49 to 0.81) was found to yield the best OS benefit when compared with chemotherapy. Meanwhile, serplulimab had the highest probability (46.11%) for better OS. Furthermore, compared with chemotherapy, serplulimab significantly increased the OS rate from the 6th to the 21st month. With regard to PFS, serplulimab (HR = 0.47, 95% CI: 0.38 to 0.59) was found to yield the best PFS benefit when compared with chemotherapy. Simultaneously, serplulimab had the highest probability (94.48%) for better PFS. Serplulimab was also a long-lasting first-line regimen in both OS and PFS from a longitudinal perspective. In addition, there was no significant difference among the various treatment options for ORR and grade ≥3 AEs.

**Conclusion:**

Considering OS, PFS, ORR, and safety profiles, serplulimab with chemotherapy should be recommended as the best therapy for patients with ES-SCLC. Certainly, more head-to-head studies are needed to confirm these findings.

**Systematic review registration:**

https://www.crd.york.ac.uk/PROSPERO/, identifier CRD42022373291.

## Background

According to the National Cancer Institute (NCI), the rate of new cases of lung and bronchus cancer was 52.0 per 100,000 persons per year. The death rate was 35.0 per 100,000 persons per year in 2019; there were an estimated 236,740 new cases in 2022 (1). According to the estimates of the National Cancer Center (NCC) of China, approximately 549,800 newly diagnosed lung cancer cases were reported in 2016; 29.7% of all deaths from cancer were ascribed to lung cancer in men and 22.9% in women (2). Small cell lung cancer (SCLC) represents approximately 15% of all lung cancers, which was a high-grade neuroendocrine carcinoma defined by its aggressiveness, poor differentiation, and somber prognosis (3, 4). The veteran’s administration lung cancer study categorizes SCLC into limited or extensive-stage disease according to whether the disease is limited to one hemithorax in a field amenable to radiation therapy (5). Despite divergent active treatment, SCLC has a bleak prognosis, with a 5-year survival rate of only approximately 7% due to factors like a high proliferative index, a quick doubling time, and a strong propensity to metastasis (5). Throughout the course of the disease, 50% of patients with SCLC will develop central nervous system (CNS) metastasis (6, 7).

For several decades, platinum drugs (cisplatin or carboplatin) plus etoposide, namely, EP protocol, have been established as the first-line standard treatment protocol for ES-SCLC. However, because of the quick emergence of resistance, the transient benefit of therapy, and the limited efficacy of subsequent lines, the survival outcomes benefit remained poor (8–10). Although some trials in Japan demonstrated that an irinotecan-based regimen as a first-line treatment for ES-SCLC had better PFS, its OS advantage was still vague (11). Thus, the above situation compels physicians and scientists to seek better first-line treatments.

One of the most significant advancements in the treatment of cancer was immunotherapy (IO), particularly immune checkpoint inhibitors (ICIs) that obstruct co-inhibitory molecules such as programmed cell death protein-1 (PD-1) and the associated programmed death ligand 1 (PD-L1) (12–15). Clinical evidence has revealed that anti-PD-L1 monoclonal antibodies like atezolizumab and durvalumab provided additional benefits in both OS and progression-free survival (PFS) when compared with platinum-based chemotherapy as the first-line treatment for patients with ES-SCLC (16–18). The National Comprehensive Cancer Network (NCCN) SCLC panel recommended certain chemotherapy plus immunotherapy regimens as preferred alternatives for patients with ES-SCLC in 2018 (9, 19).

Undoubtedly, randomized controlled trials (RCTs) with placebo are the gold standard for determining the efficacy of novel pharmaceutical treatments (20). Until now, there have been numerous regimens treating ES-SCLC, up to now, simultaneously physicians were trapped with making clinical decisions on which regimen to choose owing to the lack of direct/indirect comparisons among those agents, urgently entailing the launch of relevant studies.

Hence, we conducted a Bayesian network meta-analysis comparing the efficacy and safety of immunotherapy combinations with chemotherapy in treating ES-SCLC to provide more evidence for clinical practice.

## Methods

We conducted this meta-analysis in accordance with the Preferred Reporting Items for Systemic Review and Meta-analyses (PRISMA) checklist (**Supplementary Table 1**). This network meta-analysis (NMA) was performed and reported in accordance with the PRISMA Extension version (PRISMA-NMA) (21). This study protocol has been registered on the international prospective register of systematic review (PROSPERO) (CRD42022373291).

### Search strategy

Databases including PubMed, Embase, Cochrane Library, Scopus, Google Scholars, and ClinicalTrials.gov, and major international conferences were searched for RCTs regarding comparing immunotherapy combinations with chemotherapy as first-line treatments for patients with advanced ES-SCLC from inception to 1 November.

The search terms included the following keywords: small cell lung carcinoma, extensive-stage, first-line, immunotherapy, PD-1, PD-L1, CTLA-4, ipilimumab, atezolizumab, durvalumab, pembrolizumab, adebrelimab, serplulimab, tiragolumab, nivolumab, randomized clinical trial, and their related MeSH terms. The detailed strategy is shown in **Supplementary Table 2**.

### Selection and eligibility criteria

Two investigators independently searched and assessed the eligibility of each study by reading the title and abstract or even the full text when necessary.

The inclusion criteria were as follows:

(1) Prospective, randomized, phase 3 or 2, controlled clinical studies.
(2) Eligible patients were newly diagnosed with treatment-naive histologically or cytologically documented ES-SCLC (American Joint Committee on Cancer, 7th edition).
(3) RCTs that used immunotherapy-based combination treatment as first-line treatment settings.
(4) RCTs that used immunotherapy-based combination treatment or placebo treatment as first-line treatment settings.

The exclusion criteria were as follows:

(1) RCTs that were based on overlapping patients.
(2) RCTs with ambiguous clinical outcomes.

Prior to the evaluation of full texts, titles and abstracts were scrutinized to ascertain eligibility. To ensure that the most recent information was included, the abstracts from all the included trials and conferences were double-checked online. Any discrepancies were resolved through discussions with the senior authors.

### Data extraction

Essential clinical characteristics extracted from the enrolled studies include the following: trial name, first author, publication sources, year of publication, sample size, patients’ age and sex distribution, smoking status, histologic type, PD-L1 expression, and Eastern Cooperative Oncology Group (ECOG) performance status score. The clinical outcomes extracted included hazard ratios (HRs) with corresponding 95% confidence intervals (95% CIs) for OS (randomization to death regardless of any causes) and PFS (randomization to the progression of any causes or death regardless of any causes). Secondary endpoints items consisted of ORR; patients were evaluated as complete response (CR) or partial response (PR) according to the criteria of RECIST version 1.1 or mWHO-best overall response rate (mWHO-BORR, proportion of patients with CR or PR per mWHO), and adverse events of grade 3 or higher (Grade ≥ 3 AEs).

### Quality assessment

The quality of the included studies was checked using the Cochrane Risk of Bias Tool in Review Manager 5.3 software (Nordic Cochrane Centre, Copenhagen, Denmark) for RCTs. The data were independently extracted by two investigators (Wenjun Li and Danbei Diwu), and any discrepancies were resolved through discussions with the senior author (Hong Wang).

### Statistical analysis

HRs and odds ratios (ORs) were generated for dichotomous variants by using GeMTC (version 0.14.3) and R (version 3.5.3). OS and PFS were reported as HR with an associated 95% CI. ORR and Grade ≥ 3 AEs were reported as OR with an associated 95% CI. As for Rstudio, we set the number of iterations to 300,000 and used the first 20,000 as a burn-in sample (the thinning interval was 10); the surfaces under the cumulative ranking curve (SUCRAs) and matrices were calculated to show pairwise comparisons among regimens on OS, PFS, ORR, and grade≥3 AEs. In addition, the software can calculate the probability that each intervention is rated as the best. Furthermore, trace and density plots as well as convergence plots were generated to determine the degree of convergence. Statistical significance was set as *p* < 0.05.

## Results

### Baseline characteristics of included studies

**Figure 1:**
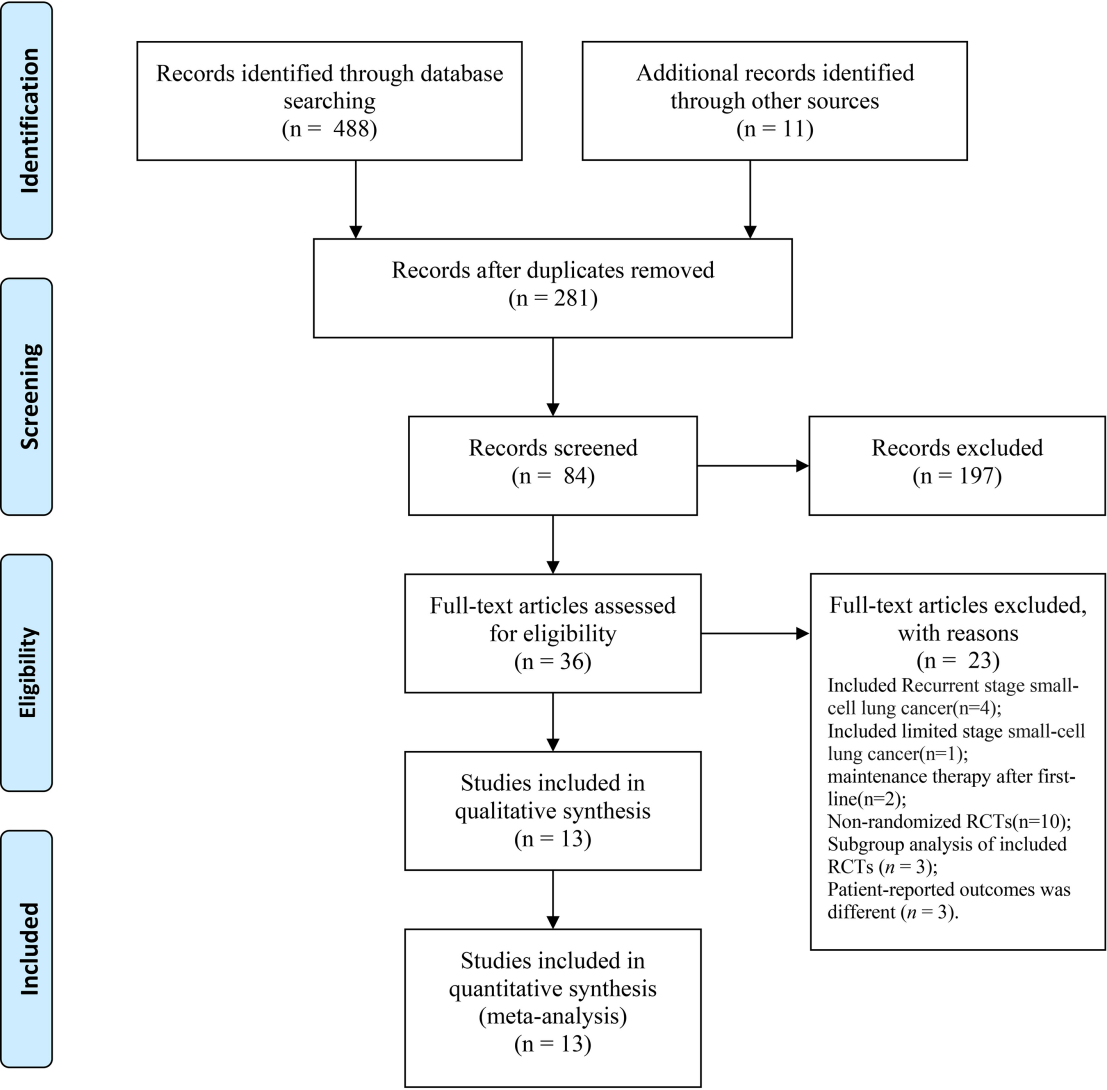
PRISMA flowchart illustrating the selection of studies included in our analyses.

**Figure 2:**
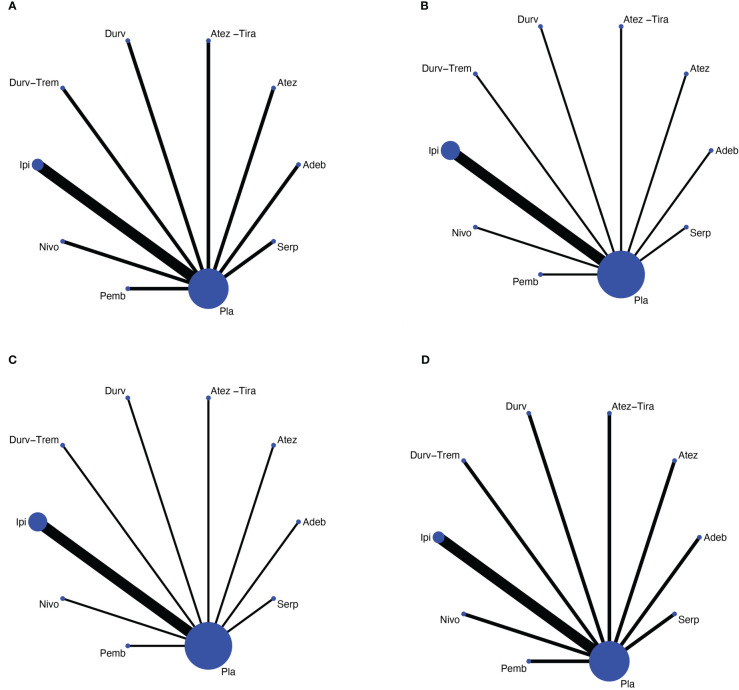
Network meta-analysis of comparisons on different outcomes of first-line treatments in different groups of ES-SCLC patients. **(A)** Comparison of overall survival (OS). **(B)** Comparison of progression-free survival (PFS). **(C)** Comparison of objective response rate (ORR). **(D)** Comparison of grade 3 or more adverse events. Direct comparisons are represented by the color lines connecting the treatments. Line width is proportional to the number of trials including every pair of treatments, whereas circle size is proportional to the total number of patients for each treatment in the network. Nivo, Nivolumab; Atez-Tira, Atezolizumab + Tiragolumab; Atez, Atezolizumab; Serp, Serplulimab; Durv, Durvalumab; Durv-Trem, Durvalumab + Tremelimumab; Pla, Placebo; Adeb, Adebrelimab; Pemb, Pembrolizumab; Ipi, Ipilimumab.

We identified a total of 488 records from the databases and 11 additional online records from the conference proceedings during the preliminary literature search. After eliminating the duplicates and non-pertinent articles through abstract screening, 13 articles finally met our eligibility criteria (**Figure 1**). A total of 4,352 individuals were enrolled to receive the following nine immunotherapy combinations across nine RCT eligible studies: ipilimumab plus etoposide/paclitaxel and platinum (Ipi); atezolizumab plus carboplatin and etoposide (Atez); durvalumab plus tremelimumab plus platinum and etoposide (Durv-Trem); durvalumab plus platinum and etoposide (Durv); pembrolizumab plus etoposide and platinum (Pemb); adebrelimab plus carboplatin and etoposide (Adeb); serplulimab plus carboplatin and etoposide (Serp); atezolizumab plus tiragolumab plus carboplatin and etoposide (Atez-Tira); and nivolumab plus carboplatin and etoposide (Nivo). Detailed information on all the included studies is presented in **Table 1**. All studies’ complete outcome reports were achieved, and 10 studies followed the principle of random allocation. All studies were at low risk of bias. The assessment of risk of bias is presented in **Supplementary Figure 1**. The network plots are depicted in **Figure 2**.

**Table 1:** Baseline characteristics of studies.

Source	Study	Phase	Treatment	Participants No.	ORR, No./total No. (%)	PFS, median, m	HR (95% CI)	*p-*value	OS, median, months	HR (95% CI)	*p*-value	Grade ≥ 3 AEs No./total No. (%)
Reck 2013	CA184-041	III	Concurrent-Ipilimumab plus paclitaxel/carboplatin	43	21/43 (48.83)	5.7 (5.2–6.9)	0.75 (0.48–1.19)	0.11	9.1 (6.7–12.9)	0.95 (0.59–1.54)	0.41	18/42 (43)
					14/43 (32.56)	3.9 (2.9–5.9)	0.93 (0.59–1.45)	0.37				
			Phased-Ipilimumab plus paclitaxel/carboplatin	42	30/42 (71.43)	6.4 (5.3–7.6)	0.64 (0.40–1.02)	0.03	12.9 (7.9–16.5)	0.75 (0.46–1.23)	0.13	21/42 (50)
					24/42 (57.14)	5.2 (4.14–6.57)	0.93 (0.59–1.48)	0.38				
			Placebo plus paclitaxel/carboplatin	45	24/45 (53.33)	5.3 (4.7–5.7)			9.9 (8.6–11.7)			13/44 (30)
Reck 2016	CA184-156	III	Ipilimumab plus etoposide and platinum	478	297/478 (62.1)	4.6 (4.5–5.0)	0.85(0.75–0.97)	0.02	11 (10.5–11.3)	0.94 (0.81–1.09)	0.377	231/478 (48.3)
			Placebo plus etoposide and platinum	476	196/476 (41.2)	4.4 (4.4–4.6)			10.9 (10–11.5)			214/476 (45.0)
Horn 2018	IMpower133	III	Atezolizumab plus carboplatin and etoposide	201	121/201 (60.2)	5.2 (4.4–5.6)	0.77(0.62–0.96)	0.02	12.3 (10.8–15.9)	0.70 (0.54–0.91)	0.007	115/198 (58.1)
			placebo plus carboplatin and etoposide	202	130/202 (64.4)	4.3 (4.2–4.5)			10.3 (9.3–11.3)			113/196 (57.7)
Ticiana 2020	EA5161	II	Nivolumab plus cisplatin/carboplatin and etoposide	80	42/80 (52.29)	5.5	0.68 (0.48–1.00)	0.047	11.3	0.73 (0.49–1.1)	0.14	67/75 (89.33)
			cisplatin/carboplatin and etoposide	80	38/80 (47.71)	4.7			9.3			50/70 (71.43)
Paz-Ares 2019-2022	CASPIAN	III	Durvalumab plus tremelimumab plus platinum-etoposide	268	156/267 (58.4)	4.9 (4.7–5.9)	0·84 (0.7–1.01)	NR	10.4 (9.5–12)	0·81 (0·67–0.97)	0·045	196/266 (73.68)
			Durvalumab plus platinum-etoposide	268	182/268 (67.9)	5.1 (4.7–6.2)	0·80(0·66–0.96)	NR	12.9 (11.3–14.7)	0.71 (0.60–0.86)	0.003	171/265 (64.53)
			Platinum-etoposide alone	269	156/269 (58.0)	5.4 (4.8–6.2)			10.5 (9.3–11.2)			173/266 (65.04)
Rudin 2022	KEYNOTE-604	III	Pembrolizumab Plus Etoposide and Platinum	228	161/228 (70.6)	4.5 (4.3–5.4)	0.75 (0.61– 0.91)	0.0023	10.8 (9.2–12.9)	0.80 (0.64–0.98)	0.016	97/223 (43.5)
			Placebo Plus Etoposide and Platinum	225	139/225 (61.8)	4.3 (4.2–4.4)			9.7 (8.6–10.7)			91/223 (40.8)
Wang 2022	CAPSTONE-1	III	Adebrelimab plus carboplatin and etoposide	230	162/230 (70.4)	5.8 (5.6–6.9)	0.67(0.54–0.83)	<0.0001	15.3 (13.2–17.5)	0.72 (0.58–0.90)	0.0017	197/230 (85.65)
			placebo plus carboplatin and etoposide	232	153/232 (65.9)	5.6 (5.5–5.7)			12.8 (11.3–13.7)			197/232 (84.91)
Cheng 2022	ASTRUM-005	III	Serplulimab plus carboplatin and etoposide	389	312/389 (80.2)	5.8 (5.5–6.9)	0.47(0.38–0.59)	<0.001	15.4 (13.3–NE)	0.63 (0.49–0.82)	<0.01	129/389 (33.2)
			placebo plus carboplatin and etoposide	196	138/196 (70.4)	4.3 (4.2–4.5)			10.9 (10–14.3)			54/196 (27.6)
Rudin 2022	SKYSCRAPER-02	III	Tiragolumab plus atezolizumab + carboplatin + etoposide	243	172/243 (70.8)	5.1 (4.4–5.4)	1.08(0.89, 1.31)	NR	13.1 (10.9–14.4)	1.02 (0.80, 1.30)	NR	166/239 (69.4)
			atezolizumab + carboplatin + etoposide	247	162/247 (65.6)	5.4 (4.5–5.7)			12.9 (12.1–14.5)			173/246 (70.3)

ES-SCLC, extensive-stage small cell lung cancer; HR, hazard ratio; NR, not reported; OS, overall survival; PFS, progression-free survival; ORR, objective response rate; Grade ≥ 3 AEs, adverse events of grade 3 or higher.

### Comparisons of OS and PFS

Nine studies were randomized studies and provided HR values for PFS and OS (**Table 2**).

**Table 2:** HR and 95% CI on 3rd, 6th, 9th, 12th, 15th, 18th, 21st, and 24th month OS for immunotherapy combinations compared to placebo.

Time (months)	Serp	Atez	Durv	Adeb	Nivo	Atez-Tira	Durv-Trem	Pemb	Ipi	Pla
3rd	1.29 (0.22,7.53)	1.31 (0.23,7.65)	1.04 (0.20,5.47)	–	2.61 (0.43,15.98)	1.36 (0.24,7.74)	–	–	–	Reference
6th	**2.02 (1.24,3.30)**	1.34 (0.78,2.29)	1.04 (0.68,1.60)	–	1.74 (0.90,3.36)	1.05 (0.61,1.79)	–	1.07 (0.69,1.64)	1.08 (0.78,1.48)	Reference
9th	**1.72 (1.19,2.50)**	1.31 (0.87,1.96)	1.42 (0.99,2.01)	0.97 (0.63,1.47)	**3.00 (1.23,7.30)**	1.09 (0.71,1.67)	1.06 (0.75,1.49)	1.14 (0.79,1.65)	1.04 (0.81,1.34)	Reference
12th	**1.66 (1.18,2.36)**	**1.60 (1.08,2.38)**	**1.71 (1.21,2.40)**	**1.66 (1.14,2.40)**	**4.03 (1.26,12.84)**	–	1.17 (0.83,1.65)	1.33 (0.92,1.94)	1.17 (0.91,1.50)	Reference
15th	**2.05 (1.44,2.92)**	**1.97 (1.30,2.97)**	**1.53 (1.07,2.18)**	**1.58 (1.09,2.28)**	16.43 (0.92,292.62)	–	1.19 (0.83,1.70)	**1.62 (1.09,2.41)**	1.18 (0.90,1.55)	Reference
18th	**1.72 (1.20,2.47)**	**1.90 (1.22,2.98)**	1.42 (0.98,2.08)	**1.95 (1.32,2.87)**	–	–	1.37 (0.94,2.00)	1.49 (0.97,2.27)	1.22 (0.89,1.67)	Reference
21st	**6.28 (3.68,10.73)**	1.52 (0.89,2.59)	1.43 (0.90,2.29)	**1.97 (1.22,3.17)**	–	–	1.42 (0.89,2.27)	**1.87 (1.10,3.18)**	1.19 (0.53,2.67)	Reference
24th	6.09 (0.68,54.14)	1.38 (0.15,12.28)	1.83 (0.21,16.16)	2.19 (0.25,19.27)	–	–	1.80 (0.20,15.84)	2.35 (0.26,21.16)	2.66 (0.26,27.37)	Reference

Nivo, nivolumab; Atez-Tira, tiragolumab; Atez, atezolizumab; Serp, serplulimab; Durv, Durvalumab; Durv-Trem, Durvalumab + tremelimumab; Pla, Placebo; Adeb, Adebrelimab; Pemb, Pembrolizumab; Ipi, ipilimumab.Significant results were in bold.

### Primary analysis: OS

**Figure 3:**
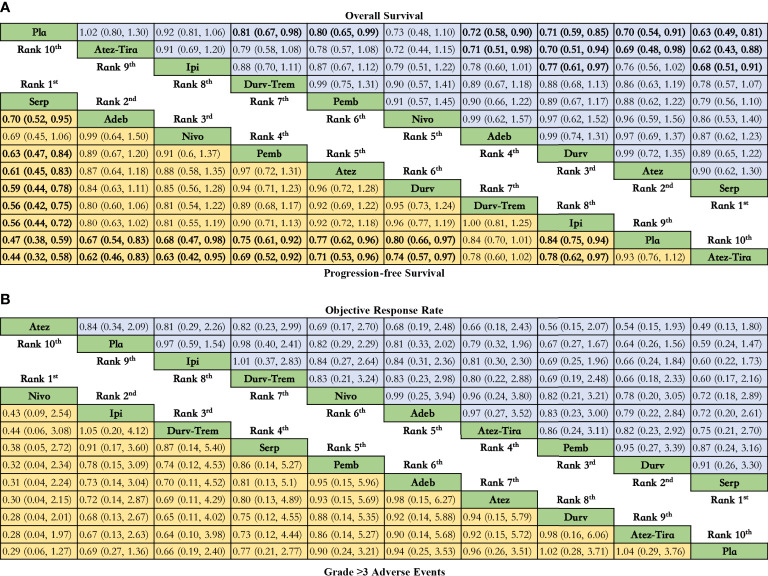
Efficacy and safety profiles of the Bayesian network meta-analysis in patients with ES-SCLS. **(A)** HRs and 95% CI for overall survival (upper triangle in blue) and progression-free survival (lower triangle in yellow), and a hazard ratio < 1.00 provides better survival benefits. **(B)** ORs and 95% CI for objective response rate (upper triangle in blue) and grade ≥ 3 adverse events (lower triangle in yellow), and an OR < 1.00 indicates a better efficacy or more toxicity. The results are presented as column-defined treatment versus row-defined treatment. Significant results are in bold. Nivo, Nivolumab; Atez-Tira, Atezolizumab + Tiragolumab; Atez, Atezolizumab; Serp, Serplulimab; Durv, Durvalumab; Durv-Trem, Durvalumab + Tremelimumab; Pla, Placebo; Adeb, Adebrelimab; Pemb, Pembrolizumab; Ipi, Ipilimumab.

**Figure 4:**
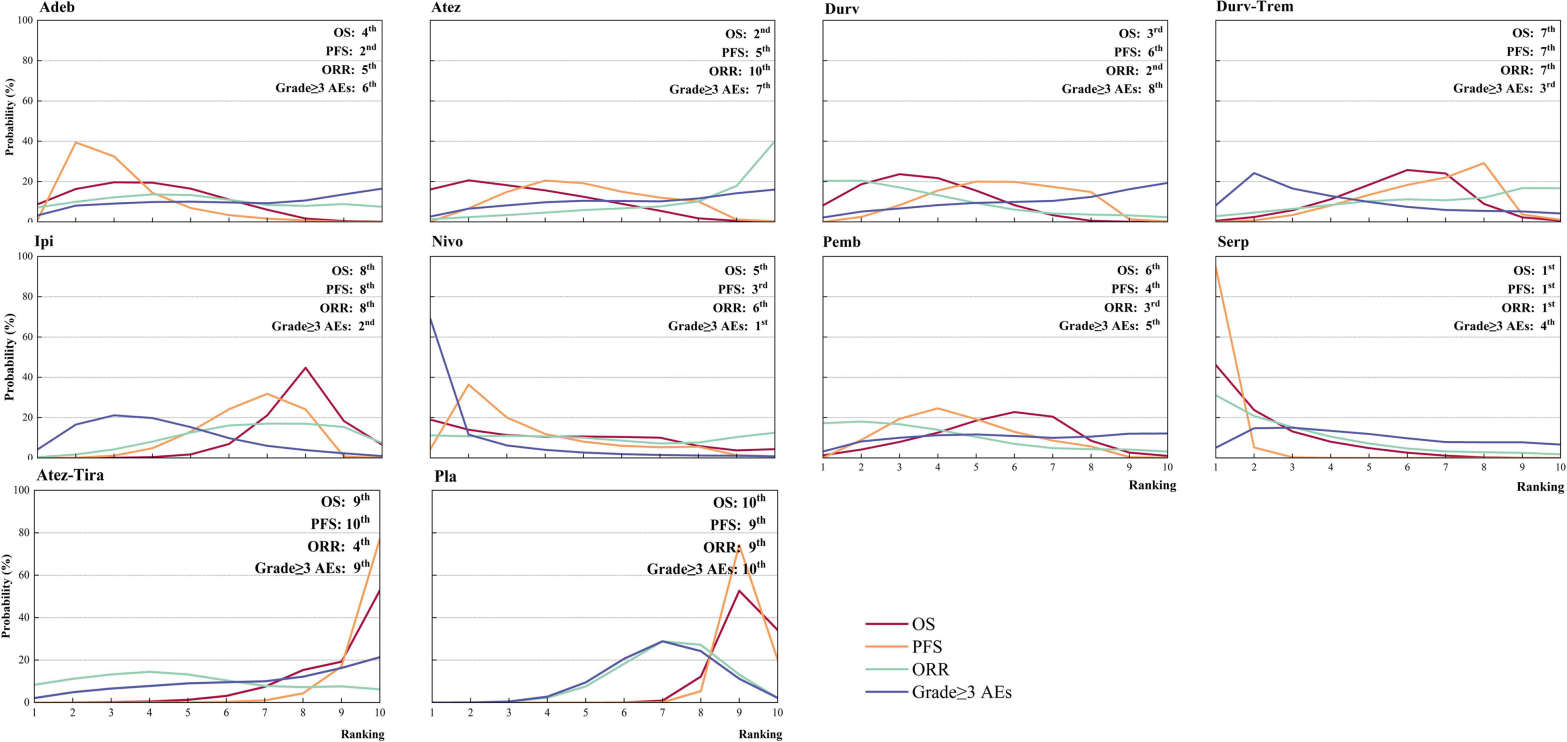
Bayesian ranking profiles for immunotherapy combinations on efficacy and safety for patients with ES-SCLC. Ranking plots indicate the probability of each comparable immunotherapy combination being ranked from first to last on OS, PFS, ORR, and grade ≥ 3 AEs. Nivo, Nivolumab; Atez-Tira, Atezolizumab + Tiragolumab; Atez, Atezolizumab; Serp, Serplulimab; Durv, Durvalumab; Durv-Trem, Durvalumab + Tremelimumab; Pla, Placebo; Adeb, Adebrelimab; Pemb, Pembrolizumab; Ipi, Ipilimumab.

Regarding OS (**Figure 3A**), compared with placebo, immunotherapy combined with chemotherapy significantly increased OS except for ipilimumab (HR = 0.92, 95% CI: 0.81 to 1.06) and atezolizumab plus tiragolumab (HR = 1.02, 95% CI: 0.80 to 1.30). Serplulimab (HR = 0.63, 95% CI: 0.49 to 0.81) was found to yield the best OS benefit when compared with placebo. Compared with ipilimumab, serplulimab (HR = 0.68, 95% CI: 0.51 to 0.91) and durvalumab (HR = 0.77, 95% CI: 0.61 to 0.97) significantly increased OS. Compared with atezolizumab plus tiragolumab, serplulimab (HR = 0.62, 95% CI: 0.43 to 0.88), atezolizumab (HR = 0.69, 95% CI: 0.48 to 0.98), durvalumab (HR = 0.70, 95% CI: 0.51-0.94), and adebrelimab (HR = 0.71, 95% CI: 0.51 to 0.98) significantly increased OS. According to Bayesian ranking profiles (**Figure 4**), serplulimab had the highest probability (46.11%) of ranking first for better OS (**Supplementary Table 4**).

**Figure 5:**
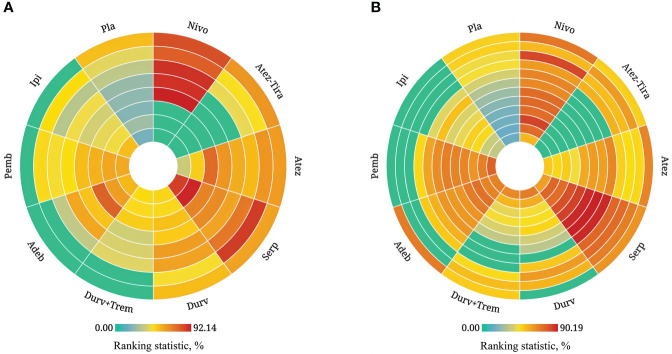
Rank-heat plot of multiple therapies in first-line treatment of patients with ES-SCLC. Each sector was colored according to the surface under the cumulative ranking (SUCRA) value of the corresponding treatment and outcome. **(A)** Rank-heat plot based on SUCRA on OS. **(B)** Rank-heat plot based on SUCRA on PFS. Circles from outside to inside refer to SUCRA value of OS on 3rd, 6th, 9th, 12th, 15th, 18th, 21st, and 24th month for immunotherapy combinations compared to chemotherapy, and SUCRA value of PFS on 1st, 2nd, 3rd, 4th, 5th, 6th, 7th, 8th, 9th, 10th, 11th, and 12th month. The closer the color is to red, the greater the probability of ranking first, and the closer the color is to green indicates 0% probability of being ranked first. Nivo, Nivolumab; Atez-Tira, Atezolizumab + Tiragolumab; Atez, Atezolizumab; Serp, Serplulimab; Durv, Durvalumab; Durv-Trem, Durvalumab + Tremelimumab; Pla, Placebo; Adeb, Adebrelimab; Pemb, Pembrolizumab; Ipi, Ipilimumab.

Regarding the OS for immunotherapy combinations compared to standard chemotherapy, the HRs at the 3rd, 6th, 9th, 12th, 15th, 18th, 21st, and 24th month were examined (**Table 2**). Compared with placebo, only serplulimab (HR = 2.02, 95% CI: 1.24 to 3.30) significantly increased the 6th month OS rate. Compared with placebo, serplulimab (HR = 1.66, 95% CI: 1.18 to 2.36), durvalumab (HR = 1.71, 95% CI: 1.21 to 2.40), adebrelimab (HR = 1.66, 95% CI: 1.14 to 2.40), atezolizumab (HR = 1.60, 95% CI: 1.08 to 2.38), and nivolumab (HR = 4.03, 95% CI: 1.26 to 12.84) significantly increased the 12th month OS rate. Compared with placebo, serplulimab (HR = 1.72, 95% CI: 1.20 to 2.47), adebrelimab (HR = 1.95, 95% CI: 1.32 to 2.87), and atezolizumab (HR = 1.90, 95% CI: 1.22 to 2.98) significantly increased the 18th month OS rate. However, there was no significant difference in efficacy among all regimens in the 24th month. The first−echelon regimens were compared to placebo from a longitudinal perspective. With regard to OS, serplulimab, atezolizumab, and durvalumab were first-echelon regimens in the 3rd to 24th month. These data were summarized based on a matrix plot of each pairwise comparison of all regimens on the efficacy across all regimens from the 3rd to 24th months (**Supplementary Table 6**). Concurrently, it could be seen from the Rank-Heat Plot that each sector was colored according to the surface under the cumulative ranking (SUCRA) value of the corresponding treatment and outcome at each month. Serplulimab has the highest ranking based on its effect compared with the rest of the regimens at each month (**Figure 5A**).

### Primary analysis: PFS

Regarding PFS (**Figure 3A**), compared with placebo, immunotherapy combined with chemotherapy significantly increased PFS except durvalumab plus tremelimumab (HR = 0.84, 95% CI: 0.70 to 1.01) and atezolizumab plus tiragolumab (HR = 1.08, 95% CI: 0.89 to 1.31). Serplulimab (HR = 0.47, 95% CI: 0.38 to 0.59) was found to yield the best PFS benefit when compared with placebo. Compared with adebrelimab (HR = 0.70, 95% CI: 0.52 to 0.95), pembrolizumab (HR = 0.63, 95% CI: 0.47 to 0.84), atezolizumab (HR = 0.61, 95% CI: 0.45 to 0.83), and durvalumab (HR = 0.59, 95% CI: 0.44 to 0.78), serplulimab significantly increased PFS. According to Bayesian ranking profiles (**Figure 4**), serplulimab had the highest probability (94.48%) of ranking first for better PFS (**Supplementary Table 4**).

From the 1st to the 4th month, there was no significant difference in efficacy among all regimens (**Table 3**). Compared with placebo, serplulimab (HR = 2.67, 95% CI: 1.27 to 5.62) barely significantly increased the 5th month PFS rate. Compared with placebo, serplulimab (HR = 3.31, 95% CI: 2.25 to 4.87), adebrelimab (HR = 1.61, 95% CI: 1.11 to 2.33), pembrolizumab (HR = 1.69, 95% CI: 1.12 to 2.55), and ipilimumab (HR = 1.32, 95% CI: 1.00 to 1.74) significantly increased the 6th month PFS rate. Compared with placebo, adebrelimab and pembrolizumab significantly increased the PFS rate from the 6th to the 12th month. In addition, from the 7th to 11th months, compared with placebo, nivolumab significantly increased the PFS rate. In contrast, the efficacy of atezolizumab plus tiragolumab was poorer than placebo from the 5th to the 12th month. The comparison was made between the first-echelon regimens and placebo from a longitudinal perspective, with regard to PFS, serplulimab, and nivolumab were first-echelon regimens at 1st to 12th month, synchronously, it was the most long-lasting regimen in the first-echelon in PFS. On the other hand, adebrelimab was also a first-echelon regimen compared with placebo at the 1st and the 4th to the 12th month in PFS. These data were summarized based on a matrix plot of each pairwise comparison of all regimens on the efficacy across all regimens from the 1st to 12th months (**Supplementary Table 7**). Concurrently, it could be seen from the Rank-Heat Plot that serplulimab and nivolumab have a higher ranking based on their effect compared with the rest of the regimens at each month (**Figure 5B**).

**Table 3:** HR and 95% CI on 1st, 2nd, 3rd, 4th, 5th, 6th, 7th, 8th, 9th, 10th, 11th, and 12th month PFS for immunotherapy combinations compared to placebo.

Time (months)	Serp	Atez	Durv	Adeb	Nivo	Atez-Tira	Durv-Trem	Pemb	Ipi	Pla
1st	1.99 (0.12,31.98)	1.99 (0.18,22.10)	–	1.99(0.18,22.10)	2.08 (0.50,8.63)	1.77 (0.51,6.14)	–	–	–	Reference
2nd	2.21 (0.02,239.30)	–	1.27 (0.01,134.76)	–	1.22 (0.01,146.78)	1.61 (0.01,177.60)	1.14 (0.01,120.44)	–	–	Reference
3rd	1.96 (0.39,9.89)	1.03 (0.21,5.10)	1.18 (0.12,11.27)	–	2.61 (0.44,15.56)	1.21 (0.24,6.11)	1.06 (0.22,5.09)	–	–	Reference
4th	2.09 (0.28,15.58)	1.08 (0.14,8.05)	1.15 (0.16,8.48)	1.13 (0.15,8.53)	1.50 (0.19,12.07)	1.27 (0.17,9.55)	–	1.04 (0.14,7.72)	–	Reference
5th	**2.67 (1.27,5.62)**	1.54 (0.72,3.30)	–	1.29 (0.60,2.76)	1.57 (0.64,3.89)	–	–	1.35 (0.64,2.87)	1.00 (0.50,1.99)	Reference
6th	**3.31 (2.25,4.87)**	**1.56 (1.00,2.43)**	–	**1.61 (1.11,2.33)**	1.74 (0.90,3.36)	–	–	**1.69 (1.12,2.55)**	**1.32 (1.00,1.74)**	Reference
7th	**3.77 (2.52,5.63)**	**1.81 (1.10,2.96)**	1.14 (0.78,1.67)	**1.81 (1.10,2.96)**	**2.40 (1.15,5.03)**	–	1.08 (0.74,1.59)	**1.81 (1.10,2.96)**	**1.19 (0.88,1.60)**	Reference
8th	**3.92 (2.54,6.05)**	**1.98 (1.16,3.39)**	**1.61 (1.08,2.41)**	**2.26 (1.47,3.46)**	**3.00 (1.23,7.30)**	–	1.34 (0.89,2.02)	**2.48 (1.48,4.16)**	**1.31 (0.95,1.82)**	Reference
9th	**3.56 (2.26,5.61)**	**1.58 (0.90,2.80)**	**1.63 (1.04,2.55)**	**2.68 (1.63,4.41)**	**3.03 (1.19,7.72)**	–	**1.59 (1.02,2.49)**	**2.52 (1.38,4.61)**	**1.59 (1.09,2.32)**	Reference
10th	**3.51 (2.20,5.62)**	**1.98 (1.06,3.72)**	**1.78 (1.11,2.87)**	**3.42 (1.91,6.15)**	**5.13 (1.64,16.02)**	–	**1.66 (1.02,2.68)**	**2.71 (1.44,5.10)**	**1.80 (1.20,2.71)**	Reference
11th	**4.02 (2.42,6.69)**	**2.10 (1.09,4.05)**	**2.64 (1.54,4.55)**	**3.34 (1.85,6.00)**	**4.03 (1.26,12.84)**	–	**2.51 (1.46,4.33)**	**3.34 (1.85,6.00)**	1.47 (0.96,2.23)	Reference
12th	**3.21 (1.92,5.37)**	**2.47 (1.18,5.16)**	**3.97 (2.13,7.40)**	**3.89 (2.07,7.31)**	2.65 (0.89,7.90)	–	**3.68 (1.97,6.87)**	**4.90(2.11,11.38)**	1.42 (0.88,2.28)	Reference

Nivo, nivolumab; Atez-Tira, tiragolumab; Atez, atezolizumab; Serp, serplulimab; Durv, Durvalumab; Durv-Trem, Durvalumab + tremelimumab; Pla, Placebo; Adeb, Adebrelimab; Pemb, Pembrolizumab; Ipi, ipilimumab.Significant results were in bold.

### Comparisons of ORR

Regarding ORR (**Figure 3B**), compared with placebo, except atezolizumab (HR = 1.19, 95% CI: 0.48 to 2.97), immunotherapy combined with chemotherapy non-significantly increased ORR. Here, compared with placebo, atezolizumab plus tiragolumab (HR = 0.79, 95% CI: 0.32 to 1.96) non-significantly increased ORR. According to Bayesian ranking profiles (**Figure 4**), serplulimab had the highest probability (31.09%) of ranking first for better ORR (**Supplementary Table 4**).

### Comparisons of safety and toxicity

Compared with placebo, the immunotherapy combined with chemotherapy elevated the toxicity whereas there was no significant difference between the various treatment options for safety and toxicity (**Figure 3B**). According to Bayesian ranking profiles, the following analysis was conducted (**Figure 4**), nivolumab had the highest probability (69.06%) of ranking first of being the most toxicity treatment for patients (**Supplementary Table 4**). AEs with a grade greater than or equal to 3 that were frequently reported for the immunotherapy combinations included neutropenia, leukopenia, thrombocytopenia, anemia, diarrhea, vomiting, decreased, appetite, nausea, fatigue, rash, pruritus, alopecia, constipation, hypothyroidism, hyperthyroidism, and pneumonitis (**Supplementary Table 5**). In the chemotherapy plus ipilimumab arm, there were five treatment-related deaths, one from liver toxicity.

### Subgroup analysis based on CNS status

**Figure 6:**
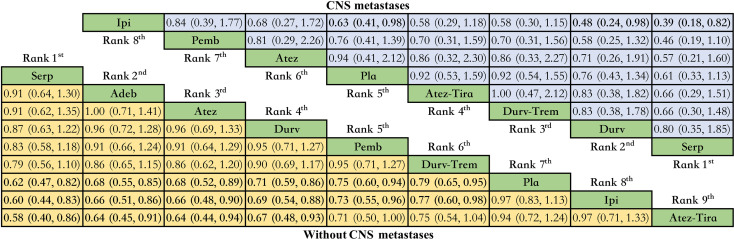
Bayesian network meta-analysis in patients with ES-SCLC. Hazard ratios and 95% CIs for patients with the baseline CNS metastases (upper triangle in blue) and for patients without baseline CNS metastases (lower triangle in yellow), and a hazard ratio < 1.00 provides better survival benefits. Hazard ratios less than 1 favor row defining treatment. Significant results are in bold. Nivo, Nivolumab; Atez-Tira, Atezolizumab + Tiragolumab; Atez, Atezolizumab; Serp, Serplulimab; Durv, Durvalumab; Durv-Trem, Durvalumab + Tremelimumab; Pla, Placebo; Adeb, Adebrelimab; Pemb, Pembrolizumab; Ipi, Ipilimumab.

Only OS network meta-analysis could be carried out, and it involved eight immunotherapy combinations for patients without CNS metastases at baseline and seven immunotherapy combinations for patients with CNS metastases (**Supplementary Table 8**). We did not have enough data to perform a meta-analysis on PFS in the subgroup of brain metastases. There was no significant difference between the various treatment options for patients with CNS metastases. In contrast, compared with placebo, serplulimab (HR = 0.62, 95% CI: 0.47 to 0.82), adebrelimab (HR = 0.68, 95% CI: 0.55 to 0.85), atezolizumab (HR = 0.68, 95% CI: 0.52 to 0.89), durvalumab (HR = 0.71, 95% CI: 0.59 to 0.86), pembrolizumab (HR = 0.75, 95% CI: 0.60 to 0.94), and durvalumab plus tremelimumab (HR = 0.79, 95% CI: 0.65 to 0.95) significantly increased OS for patients without CNS metastases (**Figure 6**).

## Discussion

To the best of our knowledge, this was the first network meta-analysis to compare the relative efficacy of all current available first-line immunotherapy combinations for ES-SCLC, which is more comprehensive than previously published studies (22, 23). In addition, this was the first network meta-analysis to make comparisons among the first-line systemic regimens on OS and PFS in ES-SCLC by each time node.

Our analysis results indicated that the immunotherapy–chemotherapy combination strategy showed significant efficacy for OS compared with placebo, except ipilimumab and tiragolumab plus atezolizumab. According to Bayesian ranking profiles, serplulimab had the highest probability for better OS, followed by atezolizumab and durvalumab, with the same results as before (23). In addition, we proved for the first time that among the first−echelon regimens compared to placebo from a longitudinal perspective, serplulimab, atezolizumab, and durvalumab were first-echelon regimens at the 3rd to 24th month on OS. These findings indicated that they may be related to better long-term survival benefits of patients with ES-SCLC. As for PFS, the immunotherapy combinations revealed better PFS than chemotherapy. The only exception was also tiragolumab plus atezolizumab, which was found to have the worst PFS of all treatments. According to Bayesian ranking profiles, serplulimab had the highest probability for better PFS, with the same results as before (23). Furthermore, in our study, serplulimab and nivolumab were first-echelon drugs from the 1st to the 12th month in PFS and had a faster onset of action compared with placebo.

In this study, efficacy and safety were well balanced in the serplulimab group, which ranked first for OS, PFS, and ORR, and fourth for grade greater than or equal to 3 AEs across all immunotherapy combinations. Serplulimab recently became the first anti-PD-1 antibody, when combined with chemotherapy, demonstrates significant improvement in the survival rates of patients with ES-SCLC (24, 25). According to our research results, serplulimab could be a first-echelon regimen because, first, it takes effect sooner and, second, the patients who benefit from it can experience long-lasting effects. Recently, serplulimab received its first approval in China for the treatment of adult patients with advanced unresectable or metastatic microsatellite instability-high (MSI-H) solid tumors that have failed to respond to previous standard treatments (26). Prior to our study, PD-L1 inhibitors might be preferred for patients with ES-SCLC, and atezolizumab and durvalumab were approved by Food and Drug Administration (FDA) as first-line treatment for patients with ES-SCLC based on the primary data from IMpower133 (27, 28) and CASPIAN (29, 30). Our study also found that the addition of atezolizumab to chemotherapy was associated with the best benefit in survival outcomes but not in ORR, with the same results as before (23). The ORR of the atezolizumab and placebo was 60.2% vs. 64.4%, respectively (17). In addition, the 3-year OS rate of durvalumab was 17.6%, which was nearly three times higher than that of chemotherapy, and the long-term survival benefit was significant. The results showed that the combination of durvalumab and EP regimen could significantly improve the OS of patients, while the combination of durvalumab and tremelimumab plus the EP regimen did not further improve the survival prognosis of ES-SCLC (10.4 vs. 10.5 months; HR = 0.81, 95% CI: 0.67 to 0.97) (31). A final analysis of a recent phase 3 clinical trial (CAPSTONE-1) showed that adebrelimab combined with carboplatin and etoposide improved the OS and PFS of ES-SCLC patients (32). In contrast, the experimental results are not very ideal for PD-1 antibody pembrolizumab and nivolumab; in terms of PFS, the efficacy of pembrolizumab and nivolumab was significantly increased compared with chemotherapy, and the secondary endpoint of OS was also improved. However, there was no significant difference between the two groups (33, 34). In terms of safety and toxicity, consistent with expectations, the immunotherapy–chemotherapy combination strategy did not observe unexpected safety events; all adverse events were controllable. A review of the included studies revealed that anti-PD-1/PD-L1 combinations with chemotherapy were relatively safe. However, toxicity increased, but remained tolerable, when anti-CTLA4/TIGIT and chemotherapy were combined (35–38). Furthermore, inclusion of nivolumab may significantly increase AEs according to our results. The PD-1 and PD-L1 antibodies were the most typical inhibitors of immunological checkpoints, with the primary function of the PD-1/PD-L1 pathway being to induce tumor cells to evade immune attacks (27). Preclinical research showed that chemotherapy altered the immune response against tumor cells and increased PD-L1 expression on tumor cells; additionally, while not reducing the number of T cells in the tumor, chemotherapy can lessen the activation and proliferation of T cells in peripheral blood (39). Head-to-head comparisons are still needed to confirm the efficacy of PD-1 and PD-L1 antibodies for patients with ES-SCLC.

According to the result of CA184-041 and CA184-156 studies, ipilimumab could significantly improve the PFS of patients with ES-SCLC; however, it could not significantly improve the OS in our study; this study confirmed the feasibility of immunotherapy combination strategy for ES-SCLC (37, 40). Ipilimumab was a monoclonal antibody (IgG1) that blocks cytotoxic T-lymphocyte-associated protein 4 (CTLA4) through its association with CD28 and enhances the T-cell response (41). SKYSCRAPER-02 evaluated the addition of tiragolumab to atezolizumab plus carboplatin and etoposide (CE), which did not provide an antitumor effect and survival benefits in patients with untreated ES-SCLC with or without brain metastases. Although the remission rate of tiragolumab was higher, it was of little significance and did not meet the prediction of ES-SCLC response rate for first-line treatment (42). Comparing Impower133 and SKYSCRAPER-02, the control arm outperformed expectations in the SKYSCRAPER-02 study, which was likely the cause of negative endpoints, in addition to the fact that an enhanced benefit in the tiragolumab arm was not seen. However, the reason for this is unclear, and further research is needed (43). TIGIT was an inhibitory receptor expressed on CD4+T cells, effector CD8+T cells, and NK cells. TIGIT interacts with CD155 expressed on antigen-presenting cells or tumor cells to downregulate T-cell and natural killer (NK) cell functions; moreover, anti-TIGIT may synergize with other immunotherapies, such as PD-L1/PD-1 inhibitors, and further amplify the immune response to improve clinical outcomes (44). However, increasing only TIGIT antibody does not appear to increase the efficacy in the tumor microenvironment where there are fewer tumor-infiltrating lymphocytes, according to some studies (45). In conclusion, adding immunosuppressive drugs to the immunological checkpoint alone does not appear to be a breakthrough in the treatment of ES-SCLC without the supervision of biomarkers.

For subgroup analysis, single metastatic sites were favorable prognostic factors in patients with ES-SCLC (5). These data support the idea that patients with asymptomatic CNS metastasis can receive first-line systemic treatment (15, 42). More ongoing clinical trials will shed further light on the safety and efficacy of immunotherapy combination with chemotherapy strategy in patients with CNS metastasis (46, 47).

Immunotherapy combination was the focus of ES-SCLC treatment, and it had higher tumor mutation load (TMB) and higher total immune cell infiltration, suggesting that it may show a greater benefit trend in immunotherapy (48, 49). Whether there were differences in tumor microenvironments between different molecular subtypes of SCLC is also a matter of concern. Recently, some real-world research findings with large sample sizes have further validated the notion that the differential expression of immune genes and predictive biomarkers in various SCLC subtypes might serve as vulnerable areas where rational and personalized treatment strategies can be targeted (50, 51). At the same time, increasing lines of evidence prove that SCLC has different cell origins, suggesting that SCLC was a heterogeneous disease. It might be a feasible strategy to improve the treatment dilemma of SCLC by molecular typing of SCLC through differences in molecular expression, exploring the characteristics of the tumor microenvironment of different molecular subtypes of SCLC, and formulating accurate treatment (50, 52). Therefore, patients with SCLC still urgently need therapeutic drugs with different mechanisms of action. In the realm of future exploration, a crucial direction lies in establishing an organic connection between key factors of SCLC molecular typing and tumor evolution. This could be accomplished through comprehensive multi-group research, aiming to identify targeted treatment strategies.

### Limitation

First, we came up with a very comprehensive search strategy; however, regrettably, publication bias limitations could have resulted from missing unpublished literature.

Second, owing to the limited number of studies that met our inclusion criteria, the inclusion of eligible studies with small sample sizes presumably increased the overall uncertainty of our results.

Third, patients were not stratified according to factors like race, which might modify treatment benefits, and the efficacy of immunotherapy combined with chemotherapy in the Asian population may differ from that in the Western population. Subsequent studies should investigate the relative treatment efficacy according to these clinical characteristics.

## Conclusion

According to our findings from this research, serplulimab combined with standard chemotherapy appears to be the best course of treatment. More head-to-head clinical trials are needed to confirm these findings.

## Data availability statement

The original contributions presented in the study are included in the article/**Supplementary Material**. Further inquiries can be directed to the corresponding author.

## Author contributions

TZ wrote the manuscript and contributed to the data analysis and interpretation. WL and DD extracted data and ensured the accuracy of the data analysis. LC and XC contributed to the data interpretation and critical revision of the manuscript for important intellectual content. HW obtained funding and approved the final version of the manuscript. All authors contributed to the article and approved the submitted version.
