# Supplementary material for: Efficacy and safety of first-line immunotherapy plus chemotherapy in treating patients with extensive-stage small cell lung cancer: a Bayesian network meta-analysis
Source: Front Immunol. 2023 Jun 26;14:1197044. doi: 10.3389/fimmu.2023.1197044 (PMC10331819; doi:10.3389/fimmu.2023.1197044)
Supplement: Supplementary file 1 [file DataSheet_1.docx]

**Efficacy and safety of first-line therapy immunotherapy plus chemotherapy in treating patients with extensive-stage small cell lung cancer: a Bayesian network meta-analysis.**

Supplementary Materials

| **Table of Contents** | | |
| --- | --- | --- |
| Title | Content | Page |
| Table S1 | Checklist of the PRISMA extension for network meta-analysis. | 2-5 |
| Table S2 | Literature search strategy. | 6 |
| Table S3 | Inconsistency Analysis of Network Meta-analysis Results. | 7 |
| Table S4 | Ranking Profiles in the Bayesian Network Meta-analysis. | 8-9 |
| Table S5 | Incidence of Grade≥3 Adverse Events in Each Immunotherapy Combination (%). | 10 |
| Table S6 | Matrix of pairwise comparisons of regimens on OS. | 11-14 |
| Table S7 | Matrix of pairwise comparisons of regimens on PFS. | 15-20 |
| Table S8 | Baseline Clinical and disease Characteristics of Trials Included in the Network Meta-analysis. | 21-22 |
| Figure S1 | Results of risk of bias assessment. | 23 |
| Figure S2 | Convergence of the three Markov Chain Monte Carlo (MCMC) chains established by of the history feature for OS, PFS, ORR, and grade ≥3 AE. | 24-27 |
| Figure S3 | Convergence of the three Markov Chain Monte Carlo (MCMC) chains established by of the Brooks-Gelman-Rubin diagnostic for OS, PFS, ORR, and grade ≥3 AE. | 28-29 |
| Figure S4 | Funnel plot for the detection of small sample effect. | 30 |

Table S1. Checklist of the PRISMA extension for network meta-analysis.

| **Section and Topic** | **Item #** | **Checklist item** | **Location where item is reported** |
| --- | --- | --- | --- |
| **TITLE** | | |  |
| Title | 1 | Identify the report as a systematic review. | 1 |
| **ABSTRACT** | | |  |
| Abstract | 2 | See the PRISMA 2020 for Abstracts checklist. | 1,2 |
| **INTRODUCTION** | | |  |
| Rationale | 3 | Describe the rationale for the review in the context of existing knowledge. | 3 |
| Objectives | 4 | Provide an explicit statement of the objective(s) or question(s) the review addresses. | 4 |
| **METHODS** | | |  |
| Eligibility criteria | 5 | Specify the inclusion and exclusion criteria for the review and how studies were grouped for the syntheses. | 5 |
| Information sources | 6 | Specify all databases, registers, websites, organisations, reference lists and other sources searched or consulted to identify studies. Specify the date when each source was last searched or consulted. | 5 |
| Search strategy | 7 | Present the full search strategies for all databases, registers and websites, including any filters and limits used. | 5,Supplementary Table 2 |
| Selection process | 8 | Specify the methods used to decide whether a study met the inclusion criteria of the review, including how many reviewers screened each record and each report retrieved, whether they worked independently, and if applicable, details of automation tools used in the process. | 6 |
| Data collection process | 9 | Specify the methods used to collect data from reports, including how many reviewers collected data from each report, whether they worked independently, any processes for obtaining or confirming data from study investigators, and if applicable, details of automation tools used in the process. | 6 |
| Data items | 10a | List and define all outcomes for which data were sought. Specify whether all results that were compatible with each outcome domain in each study were sought (e.g. for all measures, time points, analyses), and if not, the methods used to decide which results to collect. | 6 |
|  | 10b | List and define all other variables for which data were sought (e.g. participant and intervention characteristics, funding sources). Describe any assumptions made about any missing or unclear information. | 6 |
| Study risk of bias assessment | 11 | Specify the methods used to assess risk of bias in the included studies, including details of the tool(s) used, how many reviewers assessed each study and whether they worked independently, and if applicable, details of automation tools used in the process. | 7 |
| Effect measures | 12 | Specify for each outcome the effect measure(s) (e.g. risk ratio, mean difference) used in the synthesis or presentation of results. | 7 |
| Synthesis methods | 13a | Describe the processes used to decide which studies were eligible for each synthesis (e.g. tabulating the study intervention characteristics and comparing against the planned groups for each synthesis (item #5)). | 7,8 |
|  | 13b | Describe any methods required to prepare the data for presentation or synthesis, such as handling of missing summary statistics, or data conversions. | 7,8 |
|  | 13c | Describe any methods used to tabulate or visually display results of individual studies and syntheses. | 7,8 |
|  | 13d | Describe any methods used to synthesize results and provide a rationale for the choice(s). If meta-analysis was performed, describe the model(s), method(s) to identify the presence and extent of statistical heterogeneity, and software package(s) used. | 7,8 |
|  | 13e | Describe any methods used to explore possible causes of heterogeneity among study results (e.g. subgroup analysis, meta-regression). | 7,8 |
|  | 13f | Describe any sensitivity analyses conducted to assess robustness of the synthesized results. | 7,8 |
| Reporting bias assessment | 14 | Describe any methods used to assess risk of bias due to missing results in a synthesis (arising from reporting biases). | 7 |
| Certainty assessment | 15 | Describe any methods used to assess certainty (or confidence) in the body of evidence for an outcome. | 7 |
| **RESULTS** | | |  |
| Study selection | 16a | Describe the results of the search and selection process, from the number of records identified in the search to the number of studies included in the review, ideally using a flow diagram. | 8 |
|  | 16b | Cite studies that might appear to meet the inclusion criteria, but which were excluded, and explain why they were excluded. | 8, Fig. 1 |
| Study characteristics | 17 | Cite each included study and present its characteristics. | 8, Tables 1 |
| Risk of bias in studies | 18 | Present assessments of risk of bias for each included study. | 8,Supplementary Fig. 1 |
| Results of individual studies | 19 | For all outcomes, present, for each study: (a) summary statistics for each group (where appropriate) and (b) an effect estimate and its precision (e.g. confidence/credible interval), ideally using structured tables or plots. | Fig. 3, Fig. 4, Fig. 5, Fig. 6. |
| Results of syntheses | 20a | For each synthesis, briefly summarise the characteristics and risk of bias among contributing studies. | 9-13 |
|  | 20b | Present results of all statistical syntheses conducted. If meta-analysis was done, present for each the summary estimate and its precision (e.g. confidence/credible interval) and measures of statistical heterogeneity. If comparing groups, describe the direction of the effect. | 9-13 |
|  | 20c | Present results of all investigations of possible causes of heterogeneity among study results. | 9-13 |
|  | 20d | Present results of all sensitivity analyses conducted to assess the robustness of the synthesized results. | 9-13 |
| Reporting biases | 21 | Present assessments of risk of bias due to missing results (arising from reporting biases) for each synthesis assessed. | 9-13 |
| Certainty of evidence | 22 | Present assessments of certainty (or confidence) in the body of evidence for each outcome assessed. | 9-13 |
| **DISCUSSION** | | |  |
| Discussion | 23a | Provide a general interpretation of the results in the context of other evidence. | 13-17 |
|  | 23b | Discuss any limitations of the evidence included in the review. | 13-17 |
|  | 23c | Discuss any limitations of the review processes used. | 18,19 |
|  | 23d | Discuss implications of the results for practice, policy, and future research. | 19 |
| **OTHER INFORMATION** | | |  |
| Registration and protocol | 24a | Provide registration information for the review, including register name and registration number, or state that the review was not registered. | 5 |
|  | 24b | Indicate where the review protocol can be accessed, or state that a protocol was not prepared. | 6 |
|  | 24c | Describe and explain any amendments to information provided at registration or in the protocol. | 5 |
| Support | 25 | Describe sources of financial or non-financial support for the review, and the role of the funders or sponsors in the review. | 19 |
| Competing interests | 26 | Declare any competing interests of review authors. | 19 |
| Availability of data, code and other materials | 27 | Report which of the following are publicly available and where they can be found: template data collection forms; data extracted from included studies; data used for all analyses; analytic code; any other materials used in the review. | 19 |

PICOS = population, intervention, comparators, outcomes, study design.

Table S2. Literature search strategy.

| **Search Strategy** | | |
| --- | --- | --- |
| ((((("Small Cell Lung Carcinoma"[Mesh]) OR ((((((Small Cell Cancer Of The Lung[Title/Abstract]) OR (Oat Cell Carcinoma of Lung[Title/Abstract])) OR (Oat Cell Lung Cancer[Title/Abstract])) OR (Small Cell Lung Cancer[Title/Abstract])) OR (Carcinoma, Small Cell Lung[Title/Abstract])) OR (Small Cell Lung Carcinoma[Title/Abstract]))) AND ((((Extensive[Title/Abstract]) OR (Extensive Stage[Title/Abstract])) OR (Extensive-Stage[Title/Abstract])) OR (extensive-disease[Title/Abstract]))) AND ((((first-line[Title/Abstract]) OR (first line[Title/Abstract])) OR (1st line[Title/Abstract])) OR (1st-line[Title/Abstract]))) AND ((((((((((((immunotherapy[Title/Abstract]) OR (PD-1[Title/Abstract])) OR (PD-L1[Title/Abstract])) OR (CTLA-4[Title/Abstract])) OR (Ipilimumab[Title/Abstract])) OR (Atezolizumab[Title/Abstract])) OR (Durvalumab[Title/Abstract])) OR (Pembrolizumab[Title/Abstract])) OR (Adebrelimab[Title/Abstract])) OR (Serplulimab[Title/Abstract])) OR (tiragolumab[Title/Abstract])) OR (nivolumab[Title/Abstract]))) AND (((((randomized clinical trial[Title/Abstract]) OR (randomized[Title/Abstract])) OR (randomly[Title/Abstract])) OR (trial[Title/Abstract])) OR (placebo[Title/Abstract])) | | |
| **Search Strategy in PubMed** | | |
| #1 | Search "Small Cell Lung Carcinoma"[Mesh] | |
| #2 | Search (Small Cell Cancer Of The Lung[Title/Abstract]) OR (Oat Cell Carcinoma of Lung[Title/Abstract]) OR (Oat Cell Lung Cancer[Title/Abstract]) OR (Small Cell Lung Cancer[Title/Abstract]) OR (Carcinoma, Small Cell Lung[Title/Abstract]) OR (Small Cell Lung Carcinoma[Title/Abstract]) | |
| #3 | #1 OR #2 | |
| #4 | Search (Extensive[Title/Abstract]) OR (Extensive Stage[Title/Abstract]) OR (Extensive-Stage[Title/Abstract]) OR (extensive-disease[Title/Abstract]) | |
| #5 | Search (first-line[Title/Abstract]) OR (first line[Title/Abstract]) OR (1st line[Title/Abstract]) OR (1st-line[Title/Abstract]) |  |
| #6 | #3 AND #4 AND #5 |  |
| #7 | Search (immunotherapy[Title/Abstract]) OR (PD-1[Title/Abstract]) OR (PD-L1[Title/Abstract]) OR (CTLA-4[Title/Abstract]) OR (Ipilimumab[Title/Abstract]) OR (Atezolizumab[Title/Abstract]) OR (Durvalumab[Title/Abstract]) OR (Pembrolizumab[Title/Abstract]) OR (Adebrelimab[Title/Abstract]) OR (Serplulimab[Title/Abstract]) OR (tiragolumab[Title/Abstract]) OR (nivolumab[Title/Abstract]) |  |
| #8 | Search (randomized clinical trial[Title/Abstract]) OR (randomized[Title/Abstract]) OR (randomly[Title/Abstract]) OR (trial[Title/Abstract]) OR (placebo[Title/Abstract]) |  |
| #9 | #6 AND #7 AND #8 |  |

Table S3. Inconsistency Analysis of Network Meta-analysis Results.

| Outcome | Comparisons | Pooled Mean Difference (95% CI) | | I^2^ (%) | Outcome | Comparisons | Pooled Mean Difference (95% CI) | | I^2^ (%) |
| --- | --- | --- | --- | --- | --- | --- | --- | --- | --- |
|  |  | Pooled Pairwise | Pooled Network |  |  |  | Pooled Pairwise | Pooled Network |  |
| Overall survival | | | | | Progression-free survival | | | | |
|  | Pla vs Ipi | 0.91 (0.64, 1.2) | 0.91 (0.65, 1.2) | 0 |  | Pla vs Ipi | 0.83 (0.64, 1.0) | 0.83 (0.64, 1.0) | 0 |
|  | Pla vs Atez | 0.70 (0.42, 1.2) | 0.70 (0.42, 1.2) | - |  | Pla vs Atez | 0.77 (0.48, 1.2) | 0.77 (0.48, 1.2) | - |
|  | Pla vs Durv-Trem | 0.81 (0.50, 1.3) | 0.81 (0.50, 1.3) | - |  | Pla vs Durv-Trem | 0.84 (0.53, 1.3) | 0.84 (0.53, 1.3) | - |
|  | Pla vs Durv | 0.71 (0.44, 1.2) | 0.71 (0.44, 1.2) | - |  | Pla vs Durv | 0.80 (0.50, 1.3) | 0.80 (0.50, 1.3) | - |
|  | Pla vs Pemb | 0.80 (0.49, 1.3) | 0.80 (0.49, 1.3) | - |  | Pla vs Pemb | 0.75 (0.47, 1.2) | 0.75 (0.47, 1.2) | - |
|  | Pla vs Adeb | 0.72 (0.44, 1.2) | 0.72 (0.44, 1.2) | - |  | Pla vs Adeb | 0.67 (0.42, 1.1) | 0.67 (0.42, 1.1) | - |
|  | Serp vs Pla | 1.6 (0.95, 2.6) | 1.6 (0.95, 2.6) | - |  | Serp vs Pla | 2.1 (1.3, 3.4) | 2.1 (1.3, 3.4) | - |
|  | Pla vs Nivo | 0.91 (0.65, 1.2) | 0.91 (0.65, 1.2) | - |  | Pla vs Nivo | 0.68 (0.39, 1.2) | 0.68 (0.39, 1.2) | - |
|  | Atez-Tira vs Pla | 0.98 (0.77, 1.2) | 0.98 (0.77, 1.2) | - |  | Atez-Tira vs Pla | 0.93 (0.59, 1.5) | 0.93 (0.59, 1.5) | - |
| Objective response rate | | | | | Grade ≥3 adverse events | | | | |
|  | Pla vs Ipi | 0.97 (0.70, 1.3) | 0.97 (0.70, 1.3) | 32.2 |  | Pla vs Ipi | 0.75 (0.40, 1.2) | 0.75 (0.40, 1.2) | 35.8 |
|  | Pla vs Atez | 1.1 (0.61, 2.0) | 1.1 (0.61, 2.0) | - |  | Pla vs Atez | 0.97 (0.43, 2.2) | 0.97 (0.43, 2.2) | - |
|  | Pla vs Durv-Trem | 0.99 (0.54, 1.8) | 0.99 (0.54, 1.8) | - |  | Pla vs Durv-Trem | 0.79 (0.36, 1.8) | 0.79 (0.35, 1.8) | - |
|  | Pla vs Durv | 0.76 (0.42, 1.4) | 0.76 (0.42, 1.4) | - |  | Pla vs Durv | 1.0 (0.45, 2.3) | 1.0 (0.46, 2.3) | - |
|  | Pla vs Pemb | 0.79 (0.43, 1.4) | 0.79 (0.43, 1.4) | - |  | Pla vs Pemb | 0.91 (0.40, 2.1) | 0.91 (0.40, 2.1) | - |
|  | Pla vs Adeb | 0.88 (0.49, 1.6) | 0.88 (0.49, 1.6) | - |  | Pla vs Adeb | 0.98 (0.44, 2.2) | 0.98 (0.44, 2.2) | - |
|  | Serp vs Pla | 1.3 (0.74, 2.4) | 1.3 (0.74, 2.4) | - |  | Serp vs Pla | 1.2 (0.54. 2.8) | 1.2 (0.54, 2.9) | - |
|  | Pla vs Nivo | 0.87 (0.48, 1.6) | 0.87 (0.48, 1.6) | - |  | Pla vs Nivo | 0.57 (0.24, 1.4) | 0.57 (0.24, 1.4) | - |
|  | Atez-Tira vs Pla | 1.1 (0.56, 2.3) | 1.1 (0.56, 2.3) | - |  | Atez-Tira vs Pla | 0.98 (0.44. 2.2) | 0.98 (0.44. 2.2) | - |

Abbreviations: Nivo:nivolumab;Atez-Tira:atezolizumab+tiragolumab;Atez:atezolizumab;Serp:serplulimab;Durv:Durvalumab;Durv-Trem:Durvalumab+tremelimumab; Pla:Placebo;Adeb:Adebrelimab; Pemb:Pembrolizumab; Ipi:ipilimumab.

Table S4. Ranking Profiles in the Bayesian Network Meta-analysis.

| Overall Survival | Rank Probability (%) | | | | | | | | | | |
| --- | --- | --- | --- | --- | --- | --- | --- | --- | --- | --- | --- |
| Immunotherapy | Rank 1^st^ | Rank 2^nd^ | Rank 3^rd^ | Rank 4^th^ | Rank 5^th^ | | Rank 6^th^ | Rank 7^th^ | Rank 8^th^ | Rank 9^th^ | Rank 10^th^ |
| Adeb | 0.09 | 0.16 | 0.20 | 0.19 | 0.16 | | 0.11 | 0.06 | 0.02 | 0.00 | 0.00 |
| Atez | 0.16 | 0.21 | 0.18 | 0.16 | 0.12 | | 0.09 | 0.06 | 0.02 | 0.01 | 0.00 |
| Durv | 0.08 | 0.19 | 0.24 | 0.22 | 0.16 | | 0.08 | 0.03 | 0.01 | 0.00 | 0.00 |
| Durv-Trem | 0.01 | 0.02 | 0.06 | 0.11 | 0.18 | | 0.26 | 0.24 | 0.09 | 0.02 | 0.01 |
| Ipi | 0.00 | 0.00 | 0.00 | 0.00 | 0.02 | | 0.07 | 0.21 | 0.45 | 0.18 | 0.07 |
| Nivo | 0.19 | 0.14 | 0.11 | 0.10 | 0.11 | | 0.10 | 0.10 | 0.06 | 0.04 | 0.04 |
| Pemb | 0.01 | 0.04 | 0.08 | 0.13 | 0.19 | | 0.23 | 0.20 | 0.09 | 0.03 | 0.01 |
| Pla | 0.00 | 0.00 | 0.00 | 0.00 | 0.00 | | 0.00 | 0.01 | 0.12 | 0.53 | 0.34 |
| Serp | 0.46 | 0.24 | 0.13 | 0.08 | 0.05 | | 0.03 | 0.01 | 0.00 | 0.00 | 0.00 |
| Atez-Tira | 0.00 | 0.00 | 0.00 | 0.00 | 0.01 | | 0.03 | 0.07 | 0.15 | 0.19 | 0.53 |
|  | | | | | | | | | | | |
| Progression-free Survival | | | | | | Rank Probability (%) | | | | | |
| Immunotherapy | Rank 1^st^ | Rank 2^nd^ | Rank 3^rd^ | Rank 4^th^ | Rank 5^th^ | | Rank 6^th^ | Rank 7^th^ | Rank 8^th^ | Rank 9^th^ | Rank 10^th^ |
| Adeb | 0.01 | 0.39 | 0.33 | 0.14 | 0.07 | | 0.03 | 0.02 | 0.01 | 0.00 | 0.00 |
| Atez | 0.00 | 0.07 | 0.15 | 0.20 | 0.19 | | 0.15 | 0.12 | 0.10 | 0.01 | 0.00 |
| Durv | 0.00 | 0.02 | 0.08 | 0.16 | 0.20 | | 0.20 | 0.17 | 0.15 | 0.01 | 0.00 |
| Durv-Trem | 0.00 | 0.01 | 0.03 | 0.08 | 0.13 | | 0.18 | 0.22 | 0.29 | 0.04 | 0.01 |
| Ipi | 0.00 | 0.00 | 0.01 | 0.05 | 0.13 | | 0.24 | 0.32 | 0.24 | 0.01 | 0.00 |
| Nivo | 0.04 | 0.36 | 0.20 | 0.12 | 0.08 | | 0.06 | 0.05 | 0.06 | 0.01 | 0.01 |
| Pemb | 0.00 | 0.09 | 0.19 | 0.25 | 0.19 | | 0.13 | 0.09 | 0.06 | 0.00 | 0.00 |
| Pla | 0.00 | 0.00 | 0.00 | 0.00 | 0.00 | | 0.00 | 0.00 | 0.05 | 0.74 | 0.20 |
| Serp | 0.94 | 0.05 | 0.00 | 0.00 | 0.00 | | 0.00 | 0.00 | 0.00 | 0.00 | 0.00 |
| Atez-Tira | 0.00 | 0.00 | 0.00 | 0.00 | 0.00 | | 0.00 | 0.01 | 0.04 | 0.17 | 0.77 |
|  |  |  |  |  |  | |  |  |  |  |  |
| Objective Response Rate | | | | | | Rank Probability (%) | | | | | |
| Immunotherapy | Rank 1^st^ | Rank 2^nd^ | Rank 3^rd^ | Rank 4^th^ | Rank 5^th^ | | Rank 6^th^ | Rank 7^th^ | Rank 8^th^ | Rank 9^th^ | Rank 10^th^ |
| Adeb | 0.07 | 0.10 | 0.12 | 0.14 | 0.13 | | 0.11 | 0.08 | 0.08 | 0.09 | 0.07 |
| Atez | 0.01 | 0.02 | 0.03 | 0.05 | 0.06 | | 0.07 | 0.08 | 0.10 | 0.18 | 0.40 |
| Durv | 0.20 | 0.20 | 0.17 | 0.13 | 0.09 | | 0.06 | 0.04 | 0.04 | 0.03 | 0.02 |
| Durv-Trem | 0.03 | 0.05 | 0.06 | 0.08 | 0.10 | | 0.11 | 0.11 | 0.12 | 0.17 | 0.17 |
| Ipi | 0.00 | 0.02 | 0.04 | 0.08 | 0.13 | | 0.16 | 0.17 | 0.17 | 0.15 | 0.07 |
| Nivo | 0.11 | 0.11 | 0.11 | 0.11 | 0.10 | | 0.09 | 0.07 | 0.08 | 0.10 | 0.13 |
| Pemb | 0.17 | 0.18 | 0.17 | 0.14 | 0.10 | | 0.07 | 0.05 | 0.04 | 0.04 | 0.03 |
| Pla | 0.00 | 0.00 | 0.00 | 0.02 | 0.08 | | 0.18 | 0.29 | 0.27 | 0.13 | 0.02 |
| Serp | 0.31 | 0.21 | 0.15 | 0.11 | 0.07 | | 0.05 | 0.03 | 0.03 | 0.03 | 0.02 |
| Atez-Tira | 0.08 | 0.11 | 0.13 | 0.14 | 0.13 | | 0.10 | 0.08 | 0.07 | 0.08 | 0.06 |
|  |  |  |  |  |  | |  |  |  |  |  |
| Grade ≥3 Adverse Events | | | | | | Rank Probability (%) | | | | | |
| Immunotherapy | Rank 1^st^ | Rank 2^nd^ | Rank 3^rd^ | Rank 4^th^ | Rank 5^th^ | | Rank 6^th^ | Rank 7^th^ | Rank 8^th^ | Rank 9^th^ | Rank 10^th^ |
| Adeb | 0.03 | 0.08 | 0.09 | 0.10 | 0.10 | | 0.10 | 0.09 | 0.11 | 0.14 | 0.16 |
| Atez | 0.03 | 0.07 | 0.08 | 0.10 | 0.10 | | 0.10 | 0.10 | 0.12 | 0.14 | 0.16 |
| Durv | 0.02 | 0.05 | 0.07 | 0.08 | 0.09 | | 0.10 | 0.10 | 0.12 | 0.16 | 0.19 |
| Durv-Trem | 0.08 | 0.24 | 0.17 | 0.13 | 0.10 | | 0.07 | 0.06 | 0.05 | 0.05 | 0.04 |
| Ipi | 0.04 | 0.17 | 0.21 | 0.20 | 0.15 | | 0.10 | 0.06 | 0.04 | 0.02 | 0.01 |
| Nivo | 0.69 | 0.12 | 0.06 | 0.04 | 0.03 | | 0.02 | 0.01 | 0.01 | 0.01 | 0.01 |
| Pemb | 0.03 | 0.08 | 0.10 | 0.11 | 0.12 | | 0.11 | 0.10 | 0.11 | 0.12 | 0.12 |
| Pla | 0.00 | 0.00 | 0.00 | 0.03 | 0.10 | | 0.21 | 0.29 | 0.24 | 0.11 | 0.02 |
| Serp | 0.05 | 0.15 | 0.15 | 0.13 | 0.12 | | 0.10 | 0.08 | 0.08 | 0.08 | 0.07 |
| Atez-Tira | 0.02 | 0.05 | 0.07 | 0.08 | 0.09 | | 0.10 | 0.10 | 0.12 | 0.16 | 0.21 |

Abbreviations: Nivo:nivolumab;Atez-Tira:atezolizumab+tiragolumab;Atez:atezolizumab;Serp:serplulimab;Durv:Durvalumab;Durv-Trem:Durvalumab+tremelimumab; Pla:Placebo;Adeb:Adebrelimab; Pemb:Pembrolizumab; Ipi:ipilimumab.

Table S5. Incidence of Grade≥3 Adverse Events in Each Immunotherapy Combination(%).

|  | Ipi | Atez | Durv-Trem | Durv | Pemb | Adeb | Serp | Nivo | Atez -Tira | Ipi | Ipi |
| --- | --- | --- | --- | --- | --- | --- | --- | --- | --- | --- | --- |
| Neutropenia | 24 | 36.3 | 43 | 42 | 57 | 95 | 19.8 | 47 | 28 | 50 | 49 |
| Leukopenia | 6 | 7.6 | 12 | 15 | 22.4 | 94 | 20 | 5 | NR | NR | NR |
| Thrombocytopenia | 9 | 16.2 | 20 | 16 | 26.5 | 83 | 15.2 | 18 | NR | 57 | 47 |
| Anemia | 24 | 38.3 | 38 | 38 | 48.4 | 84 | 21.6 | 20 | 39 | 91 | 92 |
| Diarrhea | 25 | 9.6 | 17 | 11 | 21.1 | NR | NR | 3 | NR | 34 | 25 |
| Vomiting | 10 | 13.6 | 16 | 15 | 16.1 | 26 | NR | NR | NR | NR | NR |
| Decreased appetite | 12 | 20.7 | 22 | 18 | 30.9 | 29 | NR | NR | 22 | 10 | 19 |
| NRusea | 23 | 31.8 | 32 | 34 | 38.6 | 40 | 12.9 | NR | 25 | 29 | 24 |
| Fatigue | 13 | 21.2 | 20 | 19 | 27.4 | NR | NR | 12 | 24 | 29 | 36 |
| Rash | 19 | 20.2 | 13 | 6 | 13.5 | NR | NR | NR | 13 | 24 | 26 |
| Pruritus | 12 | NR | 15 | 8 | 11.2 | NR | NR | NR | NR | 19 | 24 |
| Alopecia | 5 | 34.8 | 30 | 32 | 33.6 | 44 | NR | NR | 23 | 67 | 57 |
| Constipation | NR | 10.1 | 21 | 17 | 29.6 | 17 | NR | NR | 23 | NR | NR |
| hypothyroidism | NR | 12.6 | NR | NR | 10.3 | 12 | 14.9 | NR | NR | NR | NR |
| hyperthyroidism | NR | 5.6 | 11 | 10 | NR | NR | 11.3 | NR | NR | NR | NR |
| pneumonitis | NR | 2.5 | 10 | 4 | 11.7 | 4 | NR | 9 | NR | NR | NR |

Abbreviations: Nivo:nivolumab;Atez-Tira:atezolizumab+tiragolumab;Atez:atezolizumab;Serp:serplulimab;Durv:Durvalumab;Durv-Trem:Durvalumab+tremelimumab; Pla:Placebo;Adeb:Adebrelimab; Pemb:Pembrolizumab; Ipi:ipilimumab; NR, not reported.

Table S6. Matrix of pairwise comparisons of regimens on OS.

| Matrix of pairwise comparisons of regimens on 3rd months OS (shown as hazard ratios and 95% confidence intervals). | | | | | | | | | | | | | | | | | | | | | | | | | | | | | | | | | | | | | |
| --- | --- | --- | --- | --- | --- | --- | --- | --- | --- | --- | --- | --- | --- | --- | --- | --- | --- | --- | --- | --- | --- | --- | --- | --- | --- | --- | --- | --- | --- | --- | --- | --- | --- | --- | --- | --- | --- |
|  | Nivo | | | Atez-Tira | | | | | | Atez | | | | | Serp | | | | Durv | | | Pla | | | Durv-Trem | | | | Adeb | | | | Pemb | | | | Ipi |
| SUCRA(%) | 81.42 | | | 63.11 | | | | | | 62.04 | | | | | 60.23 | | | | 53.91 | | | 53.23 | | | 45.76 | | | | 30.51 | | | | 28.18 | | | | 21.54 |
| Nivo | 1 | | | 0.52 (0.04,6.43) | | | | | | 0.50 (0.04,6.31) | | | | | 0.49 (0.04,6.21) | | | | 0.40 (0.03,4.66) | | | 0.38 (0.06,2.35) | | | 0.32 (0.03,3.73) | | | | 0.20 (0.02,2.70) | | | | 0.19 (0.02,2.28) | | | | 0.17 (0.01,2.02) |
| Atez-Tira | 1.92 (0.16,23.74) | | | 1 | | | | | | 0.97 (0.08,11.54) | | | | | 0.95 (0.08,11.35) | | | | 0.77 (0.07,8.51) | | | 0.74 (0.13,4.21) | | | 0.62 (0.06,6.80) | | | | 0.39 (0.03,4.93) | | | | 0.37 (0.03,4.17) | | | | 0.32 (0.03,3.68) |
| Atez | 1.98 (0.16,24.87) | | | 1.03 (0.09,12.30) | | | | | | 1 | | | | | 0.98 (0.08,11.89) | | | | 0.79 (0.07,8.92) | | | 0.76 (0.13,4.43) | | | 0.64 (0.06,7.13) | | | | 0.40 (0.03,5.16) | | | | 0.38 (0.03,4.37) | | | | 0.33 (0.03,3.86) |
| Serp | 2.02 (0.16,25.40) | | | 1.05 (0.09,12.56) | | | | | | 1.02 (0.08,12.35) | | | | | 1 | | | | 0.81 (0.07,9.11) | | | 0.78 (0.13,4.53) | | | 0.65 (0.06,7.29) | | | | 0.41 (0.03,5.28) | | | | 0.39 (0.03,4.46) | | | | 0.34 (0.03,3.94) |
| Durv | 2.50 (0.21,29.12) | | | 1.30 (0.12,14.37) | | | | | | 1.26 (0.11,14.14) | | | | | 1.24 (0.11,13.91) | | | | 1 | | | 0.96 (0.18,5.02) | | | 0.80 (0.08,8.32) | | | | 0.51 (0.04,6.05) | | | | 0.48 (0.04,5.10) | | | | 0.42 (0.04,4.51) |
| Pla | 2.61 (0.43,15.98) | | | 1.36 (0.24,7.74) | | | | | | 1.31 (0.23,7.65) | | | | | 1.29 (0.22,7.53) | | | | 1.04 (0.20,5.47) | | | 1 | | | 0.84 (0.16,4.36) | | | | 0.53 (0.08,3.35) | | | | 0.50 (0.09,2.71) | | | | 0.44 (0.08,2.41) |
| DurvTrem | 3.10 (0.27,35.97) | | | 1.62 (0.15,17.75) | | | | | | 1.56 (0.14,17.46) | | | | | 1.54 (0.14,17.18) | | | | 1.24 (0.12,12.85) | | | 1.19 (0.23,6.19) | | | 1 | | | | 0.63 (0.05,7.48) | | | | 0.59 (0.06,6.30) | | | | 0.52 (0.05,5.57) |
| Adeb | 4.93 (0.37,65.54) | | | 2.57 (0.20,32.44) | | | | | | 2.48 (0.19,31.88) | | | | | 2.44 (0.19,31.36) | | | | 1.97 (0.17,23.57) | | | 1.89 (0.30,11.98) | | | 1.59 (0.13,18.85) | | | | 1 | | | | 0.94 (0.08,11.54) | | | | 0.82 (0.07,10.19) |
| Pemb | 5.24 (0.44,62.62) | | | 2.72 (0.24,30.93) | | | | | | 2.64 (0.23,30.41) | | | | | 2.59 (0.22,29.92) | | | | 2.10 (0.20,22.41) | | | 2.01 (0.37,10.93) | | | 1.69 (0.16,17.92) | | | | 1.06 (0.09,13.01) | | | | 1 | | | | 0.87 (0.08,9.70) |
| Ipi | 5.99 (0.50,72.39) | | | 3.12 (0.27,35.77) | | | | | | 3.02 (0.26,35.17) | | | | | 2.96 (0.25,34.59) | | | | 2.40 (0.22,25.92) | | | 2.30 (0.42,12.70) | | | 1.93 (0.18,20.73) | | | | 1.22 (0.10,15.05) | | | | 1.14 (0.10,12.70) | | | | 1 |
| Matrix of pairwise comparisons of regimens on 6th months OS (shown as hazard ratios and 95% confidence intervals). | | | | | | | | | | | | | | | | | | | | | | | | | | | | | | | | | | | | | |
|  | Serp | | | Nivo | | | | | | Atez | | | | | Ipi | | | | Pemb | | | Atez-Tira | | | Durv | | | | Pla | | | | Adeb | | | | Durv-Trem |
| SUCRA(%) | 85.88 | | | 77.79 | | | | | | 63.64 | | | | | 47.16 | | | | 46.61 | | | 45.60 | | | 45.23 | | | | 40.67 | | | | 23.89 | | | | 23.52 |
| Serp | 1 | | | 0.86 (0.38,1.96) | | | | | | 0.66 (0.32,1.37) | | | | | 0.53 (0.30,0.96) | | | | 0.53 (0.28,1.01) | | | 0.52 (0.25,1.07) | | | **0.52 (0.27,0.99)** | | | | **0.50 (0.30,0.81)** | | | | **0.37 (0.17,0.81)** | | | | **0.39 (0.20,0.73)** |
| Nivo | 1.16 (0.51,2.64) | | | 1 | | | | | | 0.77 (0.33,1.80) | | | | | 0.62 (0.30,1.29) | | | | 0.61 (0.28,1.35) | | | 0.60 (0.26,1.40) | | | 0.60 (0.27,1.31) | | | | 0.57 (0.30,1.11) | | | | 0.43 (0.18,1.05) | | | | **0.45 (0.21,0.98)** |
| Atez | 1.51 (0.73,3.12) | | | 1.30 (0.56,3.04) | | | | | | 1 | | | | | 0.81 (0.43,1.50) | | | | 0.80 (0.40,1.59) | | | 0.78 (0.37,1.67) | | | 0.78 (0.39,1.55) | | | | 0.75 (0.44,1.28) | | | | 0.56 (0.25,1.25) | | | | 0.58 (0.30,1.15) |
| Ipi | 1.87 (1.04,3.36) | | | 1.61 (0.78,3.35) | | | | | | 1.24 (0.66,2.32) | | | | | 1 | | | | 0.99 (0.58,1.69) | | | 0.97 (0.52,1.81) | | | 0.97 (0.57,1.65) | | | | 0.93 (0.67,1.27) | | | | 0.70 (0.36,1.37) | | | | 0.72 (0.43,1.22) |
| Pemb | 1.89 (0.99,3.63) | | | 1.63 (0.74,3.57) | | | | | | 1.25 (0.63,2.50) | | | | | 1.01 (0.59,1.72) | | | | 1 | | | 0.98 (0.49,1.95) | | | 0.98 (0.53,1.79) | | | | 0.94 (0.61,1.44) | | | | 0.71 (0.34,1.47) | | | | 0.73 (0.40,1.33) |
| Atez-Tira | 1.93 (0.93,3.99) | | | 1.66 (0.71,3.89) | | | | | | 1.28 (0.60,2.74) | | | | | 1.03 (0.55,1.92) | | | | 1.02 (0.51,2.03) | | | 1 | | | 1.00 (0.50,1.98) | | | | 0.96 (0.56,1.63) | | | | 0.72 (0.33,1.60) | | | | 0.75 (0.38,1.47) |
| Durv | **1.94 (1.01,3.70)** | | | 1.67 (0.76,3.65) | | | | | | 1.28 (0.65,2.55) | | | | | 1.03 (0.61,1.76) | | | | 1.02 (0.56,1.87) | | | 1.00 (0.51,1.98) | | | 1 | | | | 0.96 (0.63,1.47) | | | | 0.72 (0.35,1.50) | | | | 0.75 (0.41,1.35) |
| Pla | **2.02 (1.24,3.30)** | | | 1.74 (0.90,3.36) | | | | | | 1.34 (0.78,2.29) | | | | | 1.08 (0.78,1.48) | | | | 1.07 (0.69,1.64) | | | 1.05 (0.61,1.79) | | | 1.04 (0.68,1.60) | | | | 1 | | | | 0.76 (0.42,1.36) | | | | 0.78 (0.52,1.18) |
| Adeb | **2.67 (1.24,5.76)** | | | 2.30 (0.95,5.57) | | | | | | 1.77 (0.80,3.94) | | | | | 1.43 (0.73,2.79) | | | | 1.41 (0.68,2.93) | | | 1.38 (0.62,3.07) | | | 1.38 (0.67,2.86) | | | | 1.32 (0.73,2.39) | | | | 1 | | | | 1.03 (0.50,2.13) |
| Durv-Trem | **2.58 (1.36,4.90)** | | | **2.22 (1.02,4.83)** | | | | | | 1.71 (0.87,3.37) | | | | | 1.38 (0.82,2.32) | | | | 1.37 (0.75,2.47) | | | 1.34 (0.68,2.63) | | | 1.33 (0.74,2.41) | | | | 1.28 (0.85,1.93) | | | | 0.97 (0.47,1.99) | | | | 1 |
| Matrix of pairwise comparisons of regimens on 9th months OS (shown as hazard ratios and 95% confidence intervals). | | | | | | | | | | | | | | | | | | | | | | | | | | | | | | | | | | | | | |
|  | Nivo | | | Serp | | | | | | Durv | | | | | Atez | | | | Pemb | | | Atez-Tira | | | Durv-Trem | | | | Ipi | | | | Adeb | | | | Pla |
| SUCRA(%) | 88.46 | | | 71.18 | | | | | | 60.26 | | | | | 54.86 | | | | 45.18 | | | 42.14 | | | 39.66 | | | | 33.59 | | | | 32.73 | | | | 31.94 |
| Nivo | 1 | | | 0.57 (0.22,1.50) | | | | | | 0.47 (0.18,1.23) | | | | | 0.44 (0.16,1.16) | | | | 0.38 (0.15,1.00) | | | **0.36 (0.14,0.97)** | | | **0.35 (0.14,0.91)** | | | | **0.35 (0.14,0.87)** | | | | **0.32 (0.12,0.86)** | | | | **0.33 (0.14,0.81)** |
| Serp | 1.74 (0.66,4.56) | | | 1 | | | | | | 0.82 (0.49,1.37) | | | | | 0.76 (0.44,1.31) | | | | 0.66 (0.39,1.12) | | | 0.63 (0.36,1.11) | | | 0.61 (0.37,1.02) | | | | **0.60 (0.39,0.94)** | | | | **0.56 (0.32,0.98)** | | | | **0.58 (0.40,0.84)** |
| Durv | 2.12 (0.82,5.51) | | | 1.22 (0.73,2.03) | | | | | | 1 | | | | | 0.93 (0.54,1.58) | | | | 0.81 (0.48,1.34) | | | 0.77 (0.44,1.34) | | | 0.75 (0.46,1.22) | | | | 0.74 (0.48,1.13) | | | | 0.68 (0.39,1.18) | | | | 0.71 (0.50,1.01) |
| Atez | 2.29 (0.86,6.09) | | | 1.32 (0.76,2.28) | | | | | | 1.08 (0.63,1.85) | | | | | 1 | | | | 0.87 (0.50,1.51) | | | 0.83 (0.46,1.50) | | | 0.81 (0.47,1.37) | | | | 0.80 (0.49,1.28) | | | | 0.74 (0.41,1.32) | | | | 0.76 (0.51,1.15) |
| Pemb | **2.63 (1.00,6.89)** | | | 1.51 (0.90,2.55) | | | | | | 1.24 (0.74,2.07) | | | | | 1.15 (0.66,1.99) | | | | 1 | | | 0.95 (0.54,1.68) | | | 0.93 (0.56,1.54) | | | | 0.91 (0.58,1.43) | | | | 0.85 (0.48,1.49) | | | | 0.88 (0.61,1.27) |
| Atez-Tira | **2.76 (1.03,7.40)** | | | 1.59 (0.90,2.79) | | | | | | 1.30 (0.75,2.27) | | | | | 1.21 (0.67,2.17) | | | | 1.05 (0.60,1.85) | | | 1 | | | 0.97 (0.56,1.68) | | | | 0.96 (0.58,1.57) | | | | 0.89 (0.49,1.62) | | | | 0.92 (0.60,1.41) |
| Durv-Trem | **2.84 (1.09,7.36)** | | | 1.63 (0.98,2.70) | | | | | | 1.34 (0.82,2.19) | | | | | 1.24 (0.73,2.11) | | | | 1.08 (0.65,1.79) | | | 1.03 (0.59,1.78) | | | 1 | | | | 0.99 (0.64,1.51) | | | | 0.91 (0.53,1.58) | | | | 0.95 (0.67,1.33) |
| Ipi | **2.88 (1.14,7.25)** | | | **1.66 (1.06,2.59)** | | | | | | 1.36 (0.88,2.09) | | | | | 1.26 (0.78,2.03) | | | | 1.09 (0.70,1.71) | | | 1.04 (0.64,1.71) | | | 1.01 (0.66,1.55) | | | | 1 | | | | 0.93 (0.57,1.52) | | | | 0.96 (0.75,1.23) |
| Adeb | **3.11 (1.16,8.31)** | | | **1.78 (1.02,3.13)** | | | | | | 1.47 (0.85,2.54) | | | | | 1.36 (0.76,2.43) | | | | 1.18 (0.67,2.07) | | | 1.12 (0.62,2.05) | | | 1.09 (0.63,1.89) | | | | 1.08 (0.66,1.76) | | | | 1 | | | | 1.04 (0.68,1.58) |
| Pla | **3.00 (1.23,7.30)** | | | **1.72 (1.19,2.50)** | | | | | | 1.42 (0.99,2.01) | | | | | 1.31 (0.87,1.96) | | | | 1.14 (0.79,1.65) | | | 1.09 (0.71,1.67) | | | 1.06 (0.75,1.49) | | | | 1.04 (0.81,1.34) | | | | 0.97 (0.63,1.47) | | | | 1 |
| Matrix of pairwise comparisons of regimens on 12th months OS (shown as hazard ratios and 95% confidence intervals). | | | | | | | | | | | | | | | | | | | | | | | | | | | | | | | | | | | | | |
|  | Nivo | | | Durv | | | | | | Serp | | | | | Adeb | | | | Atez | | | Pemb | | | Durv-Trem | | | | Ipi | | | | Pla | | | | Atez-Tira |
| SUCRA(%) | 88.25 | | | 61.31 | | | | | | 59.84 | | | | | 59.43 | | | | 57.62 | | | 46.48 | | | 43.47 | | | | 38.31 | | | | 24.29 | | | | 21.02 |
| Nivo | 1 | | | 0.42 (0.13,1.42) | | | | | | 0.41 (0.12,1.38) | | | | | 0.41 (0.12,1.39) | | | | 0.40 (0.12,1.35) | | | 0.33 (0.10,1.12) | | | **0.29 (0.09,0.97)** | | | | **0.29 (0.09,0.95)** | | | | **0.25 (0.08,0.79)** | | | | **0.21 (0.06,0.71)** |
| Durv | 2.36 (0.71,7.91) | | | 1 | | | | | | 0.98 (0.60,1.59) | | | | | 0.97 (0.58,1.61) | | | | 0.94 (0.56,1.58) | | | 0.78 (0.47,1.30) | | | 0.69 (0.42,1.12) | | | | 0.69 (0.45,1.05) | | | | **0.59 (0.42,0.83)** | | | | **0.50 (0.29,0.84)** |
| Serp | 2.42 (0.72,8.12) | | | 1.03 (0.63,1.67) | | | | | | 1 | | | | | 0.99 (0.60,1.66) | | | | 0.96 (0.57,1.63) | | | 0.80 (0.48,1.34) | | | 0.70 (0.43,1.15) | | | | 0.70 (0.46,1.08) | | | | **0.60 (0.42,0.85)** | | | | **0.51 (0.30,0.86)** |
| Adeb | 2.43 (0.72,8.23) | | | 1.03 (0.62,1.71) | | | | | | 1.01 (0.60,1.67) | | | | | 1 | | | | 0.97 (0.56,1.67) | | | 0.81 (0.47,1.37) | | | 0.71 (0.43,1.18) | | | | 0.71 (0.45,1.10) | | | | **0.60 (0.42,0.88)** | | | | **0.51 (0.30,0.88)** |
| Atez | 2.51 (0.74,8.56) | | | 1.06 (0.63,1.80) | | | | | | 1.04 (0.61,1.76) | | | | | 1.03 (0.60,1.78) | | | | 1 | | | 0.83 (0.48,1.43) | | | 0.73 (0.43,1.23) | | | | 0.73 (0.46,1.16) | | | | **0.62 (0.42,0.93)** | | | | **0.53 (0.30,0.92)** |
| Pemb | 3.02 (0.89,10.22) | | | 1.28 (0.77,2.13) | | | | | | 1.25 (0.75,2.08) | | | | | 1.24 (0.73,2.11) | | | | 1.20 (0.70,2.07) | | | 1 | | | 0.88 (0.53,1.46) | | | | 0.88 (0.56,1.37) | | | | 0.75 (0.52,1.09) | | | | 0.63 (0.37,1.10) |
| Durv-Trem | **3.44 (1.03,11.51)** | | | 1.45 (0.90,2.36) | | | | | | 1.42 (0.87,2.31) | | | | | 1.41 (0.85,2.34) | | | | 1.37 (0.81,2.31) | | | 1.14 (0.68,1.89) | | | 1 | | | | 1.00 (0.65,1.52) | | | | 0.85 (0.61,1.20) | | | | 0.72 (0.43,1.22) |
| Ipi | **3.45 (1.05,11.27)** | | | 1.46 (0.96,2.22) | | | | | | 1.42 (0.93,2.18) | | | | | 1.42 (0.91,2.21) | | | | 1.37 (0.86,2.18) | | | 1.14 (0.73,1.79) | | | 1.00 (0.66,1.53) | | | | 1 | | | | 0.86 (0.67,1.09) | | | | 0.72 (0.45,1.15) |
| Pla | **4.03 (1.26,12.84)** | | | **1.71 (1.21,2.40)** | | | | | | **1.66 (1.18,2.36)** | | | | | **1.66 (1.14,2.40)** | | | | **1.60 (1.08,2.38)** | | | 1.33 (0.92,1.94) | | | 1.17 (0.83,1.65) | | | | 1.17 (0.91,1.50) | | | | 1 | | | | 0.85 (0.57,1.26) |
| Atez-Tira | **4.77 (1.40,16.23)** | | | **2.02 (1.19,3.41)** | | | | | | **1.97 (1.16,3.34)** | | | | | **1.96 (1.13,3.38)** | | | | **1.90 (1.08,3.32)** | | | 1.58 (0.91,2.72) | | | 1.39 (0.82,2.34) | | | | 1.38 (0.87,2.21) | | | | 1.18 (0.79,1.76) | | | | 1 |
| Matrix of pairwise comparisons of regimens on 15th months OS (shown as hazard ratios and 95% confidence intervals). | | | | | | | | | | | | | | | | | | | | | | | | | | | | | | | | | | | | | |
|  | Nivo | | | | Serp | | | | | Atez | | | | | Pemb | | | | Adeb | | | Durv | | | Durv-Trem | | | | Ipi | | | | Pla | | | | Atez-Tira |
| SUCRA(%) | 92.14 | | | | 65.94 | | | | | 63.70 | | | | | 53.87 | | | | 52.38 | | | 50.93 | | | 37.23 | | | | 36.52 | | | | 24.55 | | | | 22.74 |
| Nivo | 1 | | | | 0.12 (0.01,2.27) | | | | | 0.12 (0.01,2.19) | | | | | 0.10 (0.01,1.81) | | | | 0.10 (0.01,1.75) | | | 0.09 (0.01,1.70) | | | 0.07 (0.00,1.32) | | | | 0.07 (0.00,1.30) | | | | 0.06 (0.00,1.08) | | | | 0.06 (0.00,1.05) |
| Serp | 8.02 (0.44,146.06) | | | | 1 | | | | | 0.96 (0.56,1.65) | | | | | 0.79 (0.47,1.35) | | | | 0.77 (0.46,1.28) | | | 0.75 (0.45,1.23) | | | **0.58 (0.35,0.96)** | | | | **0.58 (0.37,0.90)** | | | | **0.49 (0.34,0.70)** | | | | **0.46 (0.27,0.78)** |
| Atez | 8.36 (0.46,153.32) | | | | 1.04 (0.61,1.79) | | | | | 1 | | | | | 0.83 (0.47,1.46) | | | | 0.80 (0.46,1.39) | | | 0.78 (0.45,1.34) | | | 0.60 (0.35,1.04) | | | | **0.60 (0.37,0.98)** | | | | **0.51 (0.34,0.77)** | | | | **0.48 (0.27,0.85)** |
| Pemb | 10.13 (0.55,185.34) | | | | 1.26 (0.74,2.15) | | | | | 1.21 (0.68,2.14) | | | | | 1 | | | | 0.97 (0.57,1.67) | | | 0.94 (0.55,1.60) | | | 0.73 (0.43,1.25) | | | | 0.73 (0.45,1.18) | | | | **0.62 (0.41,0.92)** | | | | 0.58 (0.33,1.02) |
| Adeb | 10.42 (0.57,190.02) | | | | 1.30 (0.78,2.16) | | | | | 1.25 (0.72,2.17) | | | | | 1.03 (0.60,1.77) | | | | 1 | | | 0.97 (0.58,1.62) | | | 0.75 (0.45,1.26) | | | | 0.75 (0.48,1.18) | | | | **0.63 (0.44,0.92)** | | | | 0.60 (0.35,1.03) |
| Durv | 10.73 (0.59,195.38) | | | | 1.34 (0.81,2.21) | | | | | 1.28 (0.75,2.21) | | | | | 1.06 (0.62,1.80) | | | | 1.03 (0.62,1.72) | | | 1 | | | 0.78 (0.47,1.28) | | | | 0.77 (0.50,1.20) | | | | **0.65 (0.46,0.93)** | | | | 0.62 (0.36,1.05) |
| Durv-Trem | 13.83 (0.76,251.89) | | | | **1.72 (1.04,2.85)** | | | | | 1.65 (0.96,2.85) | | | | | 1.37 (0.80,2.33) | | | | 1.33 (0.79,2.22) | | | 1.29 (0.78,2.13) | | | 1 | | | | 1.00 (0.64,1.56) | | | | 0.84 (0.59,1.20) | | | | 0.79 (0.46,1.35) |
| Ipi | 13.89 (0.77,250.55) | | | | **1.73 (1.11,2.70)** | | | | | **1.66 (1.02,2.72)** | | | | | 1.37 (0.85,2.21) | | | | 1.33 (0.84,2.10) | | | 1.29 (0.83,2.02) | | | 1.00 (0.64,1.57) | | | | 1 | | | | 0.85 (0.65,1.11) | | | | 0.80 (0.49,1.29) |
| Pla | 16.43 (0.92,292.62) | | | | **2.05 (1.44,2.92)** | | | | | **1.97 (1.30,2.97)** | | | | | **1.62 (1.09,2.41)** | | | | **1.58 (1.09,2.28)** | | | **1.53 (1.07,2.18)** | | | 1.19 (0.83,1.70) | | | | 1.18 (0.90,1.55) | | | | 1 | | | | 0.94 (0.63,1.40) |
| Atez-Tira | 17.44 (0.95,319.33) | | | | **2.17 (1.28,3.70)** | | | | | **2.09 (1.18,3.70)** | | | | | 1.72 (0.98,3.02) | | | | 1.67 (0.97,2.88) | | | 1.62 (0.95,2.77) | | | 1.26 (0.74,2.15) | | | | 1.26 (0.78,2.03) | | | | 1.06 (0.71,1.58) | | | | 1 |
| Matrix of pairwise comparisons of regimens on 18th months OS (shown as hazard ratios and 95% confidence intervals). | | | | | | | | | | | | | | | | | | | | | | | | | | | | | | | | | | | | | |
|  | | | Adeb | | | | | | Atez | | | | Serp | | | | Pemb | | | | Durv | | | Durv-Trem | | | | Ipi | | | | Pla | | | | Atez-Tira | |
| SUCRA(%) | | | 76.48 | | | | | | 74.58 | | | | 68.10 | | | | 56.34 | | | | 52.96 | | | 49.85 | | | | 41.35 | | | | 20.75 | | | | 9.59 | |
| Adeb | | | 1 | | | | | | 0.98 (0.54,1.77) | | | | 0.88 (0.52,1.50) | | | | 0.76 (0.43,1.36) | | | | 0.73 (0.43,1.26) | | | 0.70 (0.41,1.21) | | | | 0.63 (0.38,1.03) | | | | **0.51 (0.35,0.76)** | | | | **0.38 (0.21,0.66)** | |
| Atez | | | 1.02 (0.56,1.85) | | | | | | 1 | | | | 0.90 (0.51,1.61) | | | | 0.78 (0.42,1.45) | | | | 0.75 (0.42,1.34) | | | 0.72 (0.40,1.29) | | | | 0.64 (0.37,1.11) | | | | **0.52 (0.34,0.82)** | | | | **0.39 (0.21,0.71)** | |
| Serp | | | 1.13 (0.67,1.92) | | | | | | 1.11 (0.62,1.97) | | | | 1 | | | | 0.86 (0.50,1.51) | | | | 0.83 (0.49,1.39) | | | 0.80 (0.47,1.34) | | | | 0.71 (0.44,1.14) | | | | **0.58 (0.41,0.83)** | | | | **0.43 (0.25,0.74)** | |
| Pemb | | | 1.31 (0.74,2.33) | | | | | | 1.28 (0.69,2.37) | | | | 1.16 (0.66,2.02) | | | | 1 | | | | 0.96 (0.54,1.69) | | | 0.92 (0.52,1.63) | | | | 0.82 (0.48,1.39) | | | | 0.67 (0.44,1.03) | | | | **0.49 (0.27,0.89)** | |
| Durv | | | 1.37 (0.80,2.35) | | | | | | 1.34 (0.74,2.40) | | | | 1.21 (0.72,2.03) | | | | 1.04 (0.59,1.84) | | | | 1 | | | 0.96 (0.57,1.64) | | | | 0.86 (0.52,1.40) | | | | 0.70 (0.48,1.02) | | | | **0.52 (0.30,0.90)** | |
| Durv-Trem | | | 1.42 (0.83,2.44) | | | | | | 1.39 (0.77,2.49) | | | | 1.25 (0.75,2.11) | | | | 1.08 (0.61,1.91) | | | | 1.04 (0.61,1.77) | | | 1 | | | | 0.89 (0.54,1.45) | | | | 0.73 (0.50,1.06) | | | | **0.54 (0.31,0.93)** | |
| Ipi | | | 1.60 (0.97,2.64) | | | | | | 1.56 (0.90,2.71) | | | | 1.41 (0.88,2.28) | | | | 1.22 (0.72,2.07) | | | | 1.17 (0.71,1.91) | | | 1.13 (0.69,1.84) | | | | 1 | | | | 0.82 (0.60,1.13) | | | | 0.60 (0.36,1.01) | |
| Pla | | | **1.95 (1.32,2.87)** | | | | | | **1.90 (1.22,2.98)** | | | | **1.72 (1.20,2.47)** | | | | 1.49 (0.97,2.27) | | | | 1.42 (0.98,2.08) | | | 1.37 (0.94,2.00) | | | | 1.22 (0.89,1.67) | | | | 1 | | | | 0.74 (0.49,1.11) | |
| Atez-Tira | | | **2.65 (1.51,4.66)** | | | | | | **2.59 (1.41,4.76)** | | | | **2.34 (1.36,4.04)** | | | | **2.02 (1.12,3.65)** | | | | **1.94 (1.11,3.38)** | | | **1.87 (1.07,3.26)** | | | | 1.66 (0.99,2.78) | | | | 1.36 (0.90,2.05) | | | | 1 | |
| Matrix of pairwise comparisons of regimens on 21st months OS (shown as hazard ratios and 95% confidence intervals). | | | | | | | | | | | | | | | | | | | | | | | | | | | | | | | | | | | | | |
|  | Serp | | | | | Adeb | | | | | Pemb | | | | | Atez | | | | Durv | | | Durv-Trem | | | | Ipi | | | | Pla | | | | Atez-Tira | | |
| SUCRA(%) | 90.61 | | | | | 61.56 | | | | | 59.45 | | | | | 49.96 | | | | 47.35 | | | 46.92 | | | | 43.64 | | | | 25.72 | | | | 24.79 | | |
| Serp | 1 | | | | | **0.31 (0.15,0.64)** | | | | | **0.30 (0.14,0.63)** | | | | | **0.24 (0.11,0.51)** | | | | **0.23 (0.11,0.47)** | | | **0.23 (0.11,0.46)** | | | | **0.19 (0.07,0.50)** | | | | **0.16 (0.09,0.27)** | | | | **0.14 (0.07,0.29)** | | |
| Adeb | **3.19 (1.56,6.54)** | | | | | 1 | | | | | 0.95 (0.47,1.94) | | | | | 0.77 (0.38,1.58) | | | | 0.73 (0.37,1.42) | | | 0.72 (0.37,1.41) | | | | 0.60 (0.24,1.54) | | | | **0.51 (0.32,0.82)** | | | | **0.45 (0.22,0.88)** | | |
| Pemb | **3.35 (1.58,7.12)** | | | | | 1.05 (0.51,2.14) | | | | | 1 | | | | | 0.81 (0.38,1.72) | | | | 0.77 (0.38,1.56) | | | 0.76 (0.38,1.54) | | | | 0.63 (0.24,1.67) | | | | **0.53 (0.31,0.91)** | | | | **0.47 (0.23,0.96)** | | |
| Atez | | **4.14 (1.94,8.83)** | | | | | | 1.30 (0.63,2.65) | | | | 1.24 (0.58,2.62) | | | | 1 | | | | 0.95 (0.46,1.93) | | | 0.94 (0.46,1.91) | | | | 0.78 (0.30,2.07) | | | | 0.66 (0.39,1.13) | | | | 0.58 (0.28,1.20) | | |
| Durv | | **4.38 (2.15,8.92)** | | | | | | 1.37 (0.70,2.68) | | | | 1.30 (0.64,2.65) | | | | 1.06 (0.52,2.15) | | | | 1 | | | 0.99 (0.51,1.92) | | | | 0.83 (0.33,2.11) | | | | 0.70 (0.44,1.11) | | | | 0.61 (0.31,1.21) | | |
| Durv-Trem | | **4.41 (2.17,8.97)** | | | | | | 1.38 (0.71,2.69) | | | | 1.32 (0.65,2.66) | | | | 1.06 (0.52,2.16) | | | | 1.01 (0.52,1.95) | | | 1 | | | | 0.83 (0.33,2.12) | | | | 0.70 (0.44,1.12) | | | | 0.62 (0.31,1.21) | | |
| Ipi | | **5.28 (2.00,13.94)** | | | | | | 1.65 (0.65,4.23) | | | | 1.58 (0.60,4.14) | | | | 1.28 (0.48,3.36) | | | | 1.21 (0.47,3.08) | | | 1.20 (0.47,3.05) | | | | 1 | | | | 0.84 (0.38,1.89) | | | | 0.74 (0.29,1.90) | | |
| Pla | | **6.28 (3.68,10.73)** | | | | | | **1.97 (1.22,3.17)** | | | | **1.87 (1.10,3.18)** | | | | 1.52 (0.89,2.59) | | | | 1.43 (0.90,2.29) | | | 1.42 (0.89,2.27) | | | | 1.19 (0.53,2.67) | | | | 1 | | | | 0.88 (0.54,1.43) | | |
| Atez-Tira | | **7.17 (3.46,14.84)** | | | | | | **2.24 (1.13,4.45)** | | | | **2.14 (1.04,4.41)** | | | | 1.73 (0.84,3.58) | | | | 1.64 (0.83,3.23) | | | 1.63 (0.83,3.20) | | | | 1.36 (0.53,3.49) | | | | 1.14 (0.70,1.87) | | | | 1 | | |
| Matrix of pairwise comparisons of regimens on 24th months OS (shown as hazard ratios and 95% confidence intervals). | | | | | | | | | | | | | | | | | | | | | | | | | | | | | | | | | | | | | |
|  | | | Serp | | | | Ipi | | | | | | | Pemb | | | | Adeb | | | | Durv | | | | Durv-Trem | | | | Atez | | | | Pla | | | |
| SUCRA(%) | | | 85.23 | | | | 58.02 | | | | | | | 55.27 | | | | 53.36 | | | | 47.68 | | | | 46.80 | | | | 35.87 | | | | 17.77 | | | |
| Serp | | | 1 | | | | 0.44 (0.02,10.67) | | | | | | | 0.39 (0.02,8.57) | | | | 0.36 (0.02,7.85) | | | | 0.30 (0.01,6.58) | | | | 0.30 (0.01,6.45) | | | | 0.23 (0.01,4.98) | | | | 0.16 (0.02,1.46) | | | |
| Ipi | | | 2.29 (0.09,55.93) | | | | 1 | | | | | | | 0.88 (0.04,21.78) | | | | 0.82 (0.03,19.97) | | | | 0.69 (0.03,16.73) | | | | 0.68 (0.03,16.40) | | | | 0.52 (0.02,12.67) | | | | 0.38 (0.04,3.87) | | | |
| Pemb | | | 2.59 (0.12,57.34) | | | | 1.13 (0.05,27.81) | | | | | | | 1 | | | | 0.93 (0.04,20.46) | | | | 0.78 (0.04,17.15) | | | | 0.76 (0.03,16.82) | | | | 0.59 (0.03,13.00) | | | | 0.43 (0.05,3.82) | | | |
| Adeb | | | 2.78 (0.13,60.79) | | | | 1.22 (0.05,29.51) | | | | | | | 1.08 (0.05,23.68) | | | | 1 | | | | 0.84 (0.04,18.18) | | | | 0.82 (0.04,17.83) | | | | 0.63 (0.03,13.78) | | | | 0.46 (0.05,4.03) | | | |
| Durv | | | 3.33 (0.15,72.79) | | | | 1.45 (0.06,35.32) | | | | | | | 1.29 (0.06,28.36) | | | | 1.20 (0.05,25.98) | | | | 1 | | | | 0.98 (0.05,21.35) | | | | 0.75 (0.03,16.50) | | | | 0.55 (0.06,4.83) | | | |
| Durv-Trem | | | 3.39 (0.16,74.07) | | | | 1.48 (0.06,35.94) | | | | | | | 1.31 (0.06,28.85) | | | | 1.22 (0.06,26.43) | | | | 1.02 (0.05,22.15) | | | | 1 | | | | 0.77 (0.03,16.79) | | | | 0.56 (0.06,4.91) | | | |
| Atez | | | 4.42 (0.20,97.46) | | | | 1.93 (0.08,47.27) | | | | | | | 1.71 (0.08,37.96) | | | | 1.59 (0.07,34.78) | | | | 1.33 (0.06,29.15) | | | | 1.30 (0.06,28.58) | | | | 1 | | | | 0.73 (0.08,6.48) | | | |
| Pla | | | 6.09 (0.68,54.14) | | | | 2.66 (0.26,27.37) | | | | | | | 2.35 (0.26,21.16) | | | | 2.19 (0.25,19.27) | | | | 1.83 (0.21,16.16) | | | | 1.80 (0.20,15.84) | | | | 1.38 (0.15,12.28) | | | | 1 | | | |

Abbreviations: Nivo:nivolumab;Atez-Tira:atezolizumab+tiragolumab;Atez:atezolizumab;Serp:serplulimab;Durv:Durvalumab;Durv-Trem:Durvalumab+tremelimumab; Pla:Placebo;Adeb:Adebrelimab; Pemb:Pembrolizumab; Ipi:ipilimumab.

Table S7. Matrix of pairwise comparisons of regimens on PFS.

| Matrix of pairwise comparisons of regimens on 1st months PFS (shown as hazard ratios and 95% confidence intervals). | | | | | | | | | | | | | | | | | | | |
| --- | --- | --- | --- | --- | --- | --- | --- | --- | --- | --- | --- | --- | --- | --- | --- | --- | --- | --- | --- |
|  | Nivo | Atez | | Adeb | | Serp | | Pla | | Durv-Trem | | Atez-Tira | | Durv | | Pemb | | Ipi | |
| SUCRA(%) | 69.62 | 68.67 | | 65.65 | | 62.42 | | 49.08 | | 48.43 | | 48.34 | | 42.76 | | 41.96 | | 3.06 | |
| Nivo | 1 | 0.85 (0.13,5.62) | | 0.96 (0.06,15.66) | | 0.96 (0.04,21.65) | | 0.48 (0.12,1.99) | | 0.48 (0.09,2.57) | | 0.47 (0.08,2.77) | | 0.39 (0.07,2.09) | | 0.38 (0.07,2.14) | | 0.17 (0.01,2.76) | |
| Atez | 1.18 (0.18,7.79) | 1 | | 1.13 (0.07,16.92) | | 1.13 (0.05,23.60) | | 0.57 (0.16,1.96) | | 0.56 (0.12,2.61) | | 0.55 (0.11,2.84) | | 0.46 (0.10,2.12) | | 0.44 (0.09,2.19) | | 0.20 (0.01,2.98) | |
| Adeb | 1.05 (0.06,17.12) | 0.89 (0.06,13.34) | | 1 | | 1.00 (0.03,39.43) | | 0.50 (0.05,5.58) | | 0.50 (0.04,6.52) | | 0.49 (0.04,6.81) | | 0.41 (0.03,5.30) | | 0.39 (0.03,5.33) | | 0.17 (0.01,5.29) | |
| Serp | 1.05 (0.05,23.69) | 0.89 (0.04,18.63) | | 1.00 (0.03,39.47) | | 1 | | 0.50 (0.03,8.08) | | 0.50 (0.03,9.26) | | 0.49 (0.02,9.59) | | 0.41 (0.02,7.53) | | 0.39 (0.02,7.53) | | 0.18 (0.00,6.94) | |
| Pla | 2.08 (0.50,8.63) | 1.77 (0.51,6.14) | | 1.99 (0.18,22.10) | | 1.99 (0.12,31.98) | | 1 | | 1.00 (0.41,2.43) | | 0.97 (0.34,2.83) | | 0.81 (0.33,1.98) | | 0.78 (0.29,2.14) | | 0.35 (0.03,3.90) | |
| Durv-Trem | 2.09 (0.39,11.20) | 1.77 (0.38,8.21) | | 2.00 (0.15,26.04) | | 2.00 (0.11,36.93) | | 1.00 (0.41,2.45) | | 1 | | 0.98 (0.24,3.93) | | 0.81 (0.23,2.88) | | 0.78 (0.20,3.01) | | 0.35 (0.03,4.59) | |
| Atez-Tira | 2.14 (0.36,12.64) | 1.81 (0.35,9.34) | | 2.04 (0.15,28.43) | | 2.04 (0.10,40.01) | | 1.03 (0.35,2.98) | | 1.02 (0.25,4.11) | | 1 | | 0.83 (0.21,3.35) | | 0.80 (0.19,3.47) | | 0.36 (0.03,5.01) | |
| Durv | 2.57 (0.48,13.83) | 2.19 (0.47,10.14) | | 2.46 (0.19,32.13) | | 2.46 (0.13,45.55) | | 1.24 (0.50,3.03) | | 1.23 (0.35,4.37) | | 1.20 (0.30,4.86) | | 1 | | 0.97 (0.25,3.72) | | 0.43 (0.03,5.66) | |
| Pemb | 2.66 (0.47,15.20) | 2.26 (0.46,11.20) | | 2.55 (0.19,34.60) | | 2.55 (0.13,48.83) | | 1.28 (0.47,3.50) | | 1.27 (0.33,4.89) | | 1.25 (0.29,5.40) | | 1.04 (0.27,3.98) | | 1 | | 0.45 (0.03,6.10) | |
| Ipi | 5.97 (0.36,98.52) | 5.08 (0.34,76.80) | | 5.72 (0.19,172.91) | | 5.71 (0.14,226.56) | | 2.87 (0.26,32.13) | | 2.86 (0.22,37.55) | | 2.80 (0.20,39.20) | | 2.32 (0.18,30.54) | | 2.24 (0.16,30.69) | | 1 | |
| Matrix of pairwise comparisons of regimens on 2nd months PFS (shown as hazard ratios and 95% confidence intervals). | | | | | | | | | | | | | | | | | | | |
|  | Serp | | Atez-Tira | | Durv | | Nivo | | Durv-Trem | | Pla | | Pemb | | Atez | | Ipi | | Adeb |
| SUCRA(%) | 67.92 | | 60.99 | | 54.98 | | 53.86 | | 52.04 | | 48.20 | | 46.75 | | 46.56 | | 34.36 | | 34.35 |
| Serp | 1 | | 0.73 (0.00,555.25) | | 0.58 (0.00,426.56) | | 0.55 (0.00,447.27) | | 0.51 (0.00,381.37) | | 0.45 (0.00,48.89) | | 0.42 (0.00,310.98) | | 0.42 (0.00,317.56) | | 0.28 (0.00,105.20) | | 0.25 (0.00,190.58) |
| Atez-Tira | 1.38 (0.00,1050.58) | | 1 | | 0.79 (0.00,595.43) | | 0.76 (0.00,624.21) | | 0.71 (0.00,532.34) | | 0.62 (0.01,68.65) | | 0.57 (0.00,434.08) | | 0.58 (0.00,443.25) | | 0.39 (0.00,147.10) | | 0.35 (0.00,266.00) |
| Durv | 1.74 (0.00,1288.30) | | 1.26 (0.00,950.43) | | 1 | | 0.96 (0.00,765.72) | | 0.89 (0.00,652.75) | | 0.79 (0.01,83.15) | | 0.72 (0.00,532.28) | | 0.73 (0.00,543.55) | | 0.49 (0.00,179.74) | | 0.44 (0.00,326.05) |
| Nivo | 1.82 (0.00,1480.51) | | 1.32 (0.00,1092.01) | | 1.05 (0.00,839.21) | | 1 | | 0.94 (0.00,750.30) | | 0.82 (0.01,99.26) | | 0.76 (0.00,611.76) | | 0.77 (0.00,624.63) | | 0.52 (0.00,208.85) | | 0.46 (0.00,374.86) |
| Durv-Trem | 1.94 (0.00,1438.29) | | 1.41 (0.00,1061.08) | | 1.12 (0.00,815.10) | | 1.07 (0.00,854.88) | | 1 | | 0.88 (0.01,92.79) | | 0.81 (0.00,594.25) | | 0.82 (0.00,606.84) | | 0.55 (0.00,200.64) | | 0.49 (0.00,364.00) |
| Pla | 2.21 (0.02,239.30) | | 1.61 (0.01,177.60) | | 1.27 (0.01,134.76) | | 1.22 (0.01,146.78) | | 1.14 (0.01,120.44) | | 1 | | 0.92 (0.01,98.56) | | 0.93 (0.01,101.07) | | 0.63 (0.02,23.30) | | 0.56 (0.01,60.54) |
| Pemb | 2.40 (0.00,1794.36) | | 1.75 (0.00,1323.74) | | 1.38 (0.00,1016.92) | | 1.32 (0.0,1066.41) | | 1.24 (0.00,909.17) | | 1.09 (0.01,116.18) | | 1 | | 1.01 (0.00,757.06) | | 0.68 (0.00,250.57) | | 0.61 (0.00,454.19) |
| Atez | 2.38 (0.00,1793.57) | | 1.73 (0.00,1323.13) | | 1.37 (0.00,1016.49) | | 1.31 (0.0,1065.81) | | 1.22 (0.00,908.79) | | 1.07 (0.01,116.62) | | 0.99 (0.00,741.06) | | 1 | | 0.67 (0.00,250.77) | | 0.60 (0.00,454.06) |
| Ipi | 3.53 (0.01,1308.64) | | 2.56 (0.01,967.09) | | 2.03 (0.01,740.31) | | 1.94 (0.00,784.86) | | 1.82 (0.00,661.80) | | 1.59 (0.04,59.21) | | 1.47 (0.00,540.21) | | 1.48 (0.00,552.31) | | 1 | | 0.90 (0.00,331.16) |
| Adeb | 3.93 (0.01,2948.72) | | 2.86 (0.00,2175.23) | | 2.26 (0.00,1670.37) | | 2.16 (0.0,1752.22) | | 2.03 (0.0,1493.33) | | 1.78 (0.02,191.35) | | 1.64 (0.0,1217.93) | | 1.66 (0.0,1243.88) | | 1.12 (0.00,411.90) | | 1 |
| Matrix of pairwise comparisons of regimens on 3rd months PFS (shown as hazard ratios and 95% confidence intervals). | | | | | | | | | | | | | | | | | | | |
|  | Nivo | Serp | | Durv | | Atez-Tira | | Durv-Trem | | Atez | | Pla | | Adeb | | Pemb | | Ipi | |
| SUCRA(%) | 81.50 | 73.02 | | 60.89 | | 52.89 | | 46.44 | | 45.27 | | 42.67 | | 35.94 | | 35.56 | | 25.84 | |
| Nivo | 1 | 0.75 (0.07,8.38) | | 0.55 (0.05,5.90) | | 0.46 (0.04,5.17) | | 0.41 (0.04,4.39) | | 0.39 (0.04,4.34) | | 0.38 (0.06,2.29) | | 0.32 (0.03,3.55) | | 0.33 (0.03,3.60) | | 0.25 (0.03,2.20) | |
| Serp | 1.33 (0.12,14.82) | 1 | | 1.68 (0.18,15.72) | | 0.62 (0.06,6.09) | | 0.54 (0.06,5.15) | | 0.53 (0.05,5.11) | | 0.51 (0.10,2.57) | | 0.43 (0.04,4.17) | | 0.43 (0.04,4.24) | | 0.77 (0.10,5.78) | |
| Durv | 1.83 (0.17,19.81) | 1.38 (0.14,13.17) | | 1 | | 0.85 (0.09,8.13) | | 0.75 (0.08,6.88) | | 0.72 (0.08,6.82) | | 0.70 (0.15,3.39) | | 0.60 (0.06,5.57) | | 0.60 (0.06,5.66) | | 0.46 (0.06,3.40) | |
| Atez-Tira | 2.16 (0.19,24.08) | 1.62 (0.16,16.03) | | 1.67 (0.18,15.88) | | 1 | | 0.88 (0.09,8.38) | | 0.85 (0.09,8.31) | | 0.83 (0.16,4.19) | | 0.70 (0.07,6.78) | | 0.70 (0.07,6.89) | | 0.77 (0.10,5.85) | |
| Durv-Trem | 2.46 (0.23,26.44) | 1.85 (0.19,17.57) | | 1.67 (0.18,15.88) | | 1.14 (0.12,10.85) | | 1 | | 0.97 (0.10,9.10) | | 0.94 (0.20,4.51) | | 0.80 (0.09,7.43) | | 0.80 (0.08,7.55) | | 0.64 (0.08,4.80) | |
| Atez | 2.53 (0.23,27.87) | 1.90 (0.20,18.54) | | 1.34 (0.15,12.35) | | 1.17 (0.12,11.45) | | 1.03 (0.11,9.68) | | 1 | | 0.97 (0.20,4.81) | | 0.82 (0.09,7.84) | | 0.83 (0.09,7.97) | | 0.62 (0.08,4.53) | |
| Pla | 2.61 (0.44,15.56) | 1.96 (0.39,9.89) | | 1.18 (0.12,11.27) | | 1.21 (0.24,6.11) | | 1.06 (0.22,5.09) | | 1.03 (0.21,5.10) | | 1 | | 0.85 (0.17,4.15) | | 0.85 (0.17,4.24) | | 0.54 (0.07,4.16) | |
| Adeb | 3.08 (0.28,33.63) | 2.31 (0.24,22.36) | | 0.73 (0.08,6.94) | | 1.43 (0.15,13.81) | | 1.25 (0.13,11.68) | | 1.22 (0.13,11.59) | | 1.18 (0.24,5.79) | | 1 | | 1.00 (0.10,9.61) | | 0.33 (0.04,2.56) | |
| Pemb | 3.07 (0.28,33.94) | 2.31 (0.24,22.58) | | 1.67 (0.18,15.88) | | 1.42 (0.15,13.95) | | 1.25 (0.13,11.80) | | 1.21 (0.13,11.70) | | 1.18 (0.24,5.87) | | 1.00 (0.10,9.55) | | 1 | | 0.77 (0.10,5.85) | |
| Ipi | 3.97 (0.45,34.79) | 2.99 (0.39,22.83) | | 2.17 (0.29,15.99) | | 1.84 (0.24,14.10) | | 1.62 (0.22,11.87) | | 1.57 (0.21,11.81) | | 1.52 (0.45,5.22) | | 1.29 (0.17,9.63) | | 1.29 (0.17,9.81) | | 1 | |
| Matrix of pairwise comparisons of regimens on 4th months PFS (shown as hazard ratios and 95% confidence intervals). | | | | | | | | | | | | | | | | | | | |
|  | Serp | Nivo | | Atez-Tira | | Durv | | Adeb | | Atez | | Pemb | | Pla | | Durv-Trem | | Ipi | |
| SUCRA(%) | 73.99 | 61.41 | | 55.11 | | 50.82 | | 50.30 | | 48.25 | | 46.85 | | 43.87 | | 40.79 | | 28.61 | |
| Serp | 1 | 0.72 (0.04,12.97) | | 0.61 (0.04,10.47) | | 0.55 (0.03,9.35) | | 0.54 (0.03,9.34) | | 0.51 (0.03,8.84) | | 0.50 (0.03,8.50) | | 0.48 (0.06,3.57) | | 0.43 (0.03,7.35) | | 0.31 (0.03,3.79) | |
| Nivo | 1.40 (0.08,25.36) | 1 | | 0.85 (0.05,15.49) | | 0.77 (0.04,13.84) | | 0.75 (0.04,13.81) | | 0.72 (0.04,13.08) | | 0.70 (0.04,12.59) | | 0.67 (0.08,5.40) | | 0.60 (0.03,10.88) | | 0.44 (0.03,5.65) | |
| Atez-Tira | 1.65 (0.10,28.41) | 1.18 (0.06,21.51) | | 1 | | 0.90 (0.05,15.50) | | 0.89 (0.05,15.48) | | 0.85 (0.05,14.66) | | 0.82 (0.05,14.10) | | 0.79 (0.10,5.93) | | 0.71 (0.04,12.19) | | 0.51 (0.04,6.28) | |
| Durv | 1.82 (0.11,30.99) | 1.30 (0.07,23.47) | | 1.11 (0.06,18.94) | | 1 | | 0.98 (0.06,16.89) | | 0.94 (0.05,15.99) | | 0.91 (0.05,15.38) | | 0.87 (0.12,6.43) | | 0.79 (0.05,13.30) | | 0.57 (0.05,6.84) | |
| Adeb | 1.85 (0.11,32.01) | 1.32 (0.07,24.24) | | 1.12 (0.06,19.56) | | 1.02 (0.06,17.47) | | 1 | | 0.95 (0.06,16.52) | | 0.92 (0.05,15.89) | | 0.89 (0.12,6.69) | | 0.80 (0.05,13.73) | | 0.58 (0.05,7.08) | |
| Atez | 1.94 (0.11,33.34) | 1.39 (0.08,25.24) | | 1.18 (0.07,20.37) | | 1.07 (0.06,18.19) | | 1.05 (0.06,18.17) | | 1 | | 0.97 (0.06,16.55) | | 0.93 (0.12,6.94) | | 0.84 (0.05,14.30) | | 0.61 (0.05,7.37) | |
| Pemb | 2.01 (0.12,34.26) | 1.44 (0.08,25.95) | | 1.22 (0.07,20.94) | | 1.10 (0.07,18.69) | | 1.08 (0.06,18.67) | | 1.03 (0.06,17.68) | | 1 | | 0.96 (0.13,7.12) | | 0.87 (0.05,14.70) | | 0.63 (0.05,7.57) | |
| Pla | 2.09 (0.28,15.58) | 1.50 (0.19,12.07) | | 1.27 (0.17,9.55) | | 1.15 (0.16,8.48) | | 1.13 (0.15,8.53) | | 1.08 (0.14,8.05) | | 1.04 (0.14,7.72) | | 1 | | 0.90 (0.12,6.66) | | 0.65 (0.15,2.87) | |
| Durv-Trem | 2.31 (0.14,39.27) | 1.65 (0.09,29.74) | | 1.40 (0.08,23.99) | | 1.27 (0.08,21.42) | | 1.25 (0.07,21.40) | | 1.19 (0.07,20.26) | | 1.15 (0.07,19.49) | | 1.11 (0.15,8.14) | | 1 | | 0.72 (0.06,8.67) | |
| Ipi | 3.20 (0.26,38.75) | 2.29 (0.18,29.57) | | 1.94 (0.16,23.70) | | 1.76 (0.15,21.12) | | 1.73 (0.14,21.14) | | 1.65 (0.14,20.00) | | 1.59 (0.13,19.22) | | 1.53 (0.35,6.71) | | 1.38 (0.12,16.60) | | 1 | |
| Matrix of pairwise comparisons of regimens on 5th months PFS (shown as hazard ratios and 95% confidence intervals). | | | | | | | | | | | | | | | | | | | |
|  | Serp | Nivo | | Atez | | Pemb | | Adeb | | Ipi | | Pla | | Durv | | Atez-Tira | | Durv-Trem | |
| SUCRA(%) | 88.37 | 65.59 | | 65.37 | | 58.30 | | 55.21 | | 39.18 | | 36.89 | | 34.39 | | 28.57 | | 28.13 | |
| Serp | 1 | 0.59 (0.18,1.90) | | 0.58 (0.20,1.68) | | 0.51 (0.18,1.46) | | 0.48 (0.17,1.40) | | 0.38 (0.14,1.03) | | **0.37 (0.18,0.79)** | | **0.35 (0.12,1.00)** | | **0.32 (0.11,0.92)** | | **0.32 (0.11,0.90)** | |
| Nivo | 1.70 (0.53,5.48) | 1 | | 0.98 (0.30,3.20) | | 0.86 (0.26,2.79) | | 0.82 (0.25,2.68) | | 0.64 (0.20,1.98) | | 0.64 (0.26,1.57) | | 0.59 (0.19,1.91) | | 0.54 (0.16,1.76) | | 0.54 (0.17,1.72) | |
| Atez | 1.74 (0.60,5.05) | 1.02 (0.31,3.35) | | 1 | | 0.88 (0.30,2.57) | | 0.84 (0.28,2.47) | | 0.65 (0.23,1.82) | | 0.65 (0.30,1.40) | | 0.61 (0.21,1.76) | | 0.55 (0.19,1.62) | | 0.55 (0.19,1.59) | |
| Pemb | 1.98 (0.68,5.70) | 1.16 (0.36,3.78) | | 1.14 (0.39,3.33) | | 1 | | 0.95 (0.33,2.79) | | 0.74 (0.27,2.06) | | 0.74 (0.35,1.57) | | 0.69 (0.24,1.99) | | 0.63 (0.21,1.83) | | 0.62 (0.22,1.79) | |
| Adeb | 2.07 (0.71,6.04) | 1.22 (0.37,4.00) | | 1.20 (0.41,3.53) | | 1.05 (0.36,3.08) | | 1 | | 0.78 (0.28,2.18) | | 0.78 (0.36,1.67) | | 0.73 (0.25,2.11) | | 0.66 (0.22,1.94) | | 0.66 (0.23,1.90) | |
| Ipi | 2.66 (0.97,7.34) | 1.57 (0.50,4.89) | | 1.54 (0.55,4.29) | | 1.35 (0.49,3.74) | | 1.28 (0.46,3.59) | | 1 | | 1.00 (0.50,1.99) | | 0.93 (0.34,2.56) | | 0.84 (0.30,2.36) | | 0.84 (0.31,2.31) | |
| Pla | **2.67 (1.27,5.62)** | 1.57 (0.64,3.89) | | 1.54 (0.72,3.30) | | 1.35 (0.64,2.87) | | 1.29 (0.60,2.76) | | 1.00 (0.50,1.99) | | 1 | | 0.94 (0.45,1.96) | | 0.85 (0.39,1.82) | | 0.84 (0.40,1.76) | |
| Durv | **2.85 (1.00,8.13)** | 1.68 (0.52,5.41) | | 1.64 (0.57,4.76) | | 1.44 (0.50,4.15) | | 1.37 (0.47,3.98) | | 1.07 (0.39,2.93) | | 1.07 (0.51,2.24) | | 1 | | 0.90 (0.31,2.62) | | 0.90 (0.32,2.56) | |
| Atez-Tira | **3.15 (1.08,9.18)** | 1.86 (0.57,6.09) | | 1.82 (0.62,5.37) | | 1.60 (0.55,4.68) | | 1.52 (0.51,4.49) | | 1.18 (0.42,3.31) | | 1.18 (0.55,2.54) | | 1.11 (0.38,3.20) | | 1 | | 1.00 (0.34,2.89) | |
| Durv-Trem | **3.17 (1.11,9.03)** | 1.87 (0.58,6.00) | | 1.82 (0.63,5.28) | | 1.60 (0.56,4.60) | | 1.53 (0.53,4.42) | | 1.19 (0.43,3.26) | | 1.19 (0.57,2.48) | | 1.11 (0.39,3.15) | | 1.00 (0.35,2.91) | | 1 | |
| Matrix of pairwise comparisons of regimens on 6th months PFS (shown as hazard ratios and 95% confidence intervals). | | | | | | | | | | | | | | | | | | | |
|  | Serp | Pemb | | Nivo | | Adeb | | Atez | | Ipi | | Pla | | Durv | | Durv-Trem | | Atez-Tira | |
| SUCRA(%) | 90.19 | 64.96 | | 64.25 | | 61.78 | | 59.76 | | 51.80 | | 31.61 | | 30.12 | | 26.31 | | 19.22 | |
| Serp | 1 | 0.51 (0.29,0.90) | | 0.53 (0.25,1.13) | | **0.49 (0.28,0.83)** | | **0.47 (0.26,0.85)** | | **0.40 (0.25,0.64)** | | **0.30 (0.21,0.44)** | | **0.30 (0.18,0.49)** | | **0.27 (0.16,0.45)** | | **0.23 (0.13,0.41)** | |
| Pemb | 1.96 (1.12,3.45) | 1 | | 1.03 (0.47,2.24) | | 0.95 (0.55,1.66) | | 0.92 (0.50,1.69) | | 0.78 (0.48,1.28) | | **0.59 (0.39,0.89)** | | **0.58 (0.34,0.99)** | | **0.53 (0.31,0.90)** | | **0.46 (0.26,0.82)** | |
| Nivo | 1.90 (0.89,4.08) | 0.97 (0.45,2.11) | | 1 | | 0.92 (0.43,1.97) | | 0.89 (0.40,1.98) | | 0.76 (0.37,1.55) | | 0.57 (0.30,1.11) | | 0.56 (0.27,1.18) | | 0.51 (0.24,1.08) | | **0.44 (0.20,0.96)** | |
| Adeb | **2.06 (1.21,3.51)** | 1.05 (0.60,1.83) | | 1.08 (0.51,2.30) | | 1 | | 0.97 (0.54,1.73) | | 0.82 (0.52,1.30) | | **0.62 (0.43,0.90)** | | **0.61 (0.37,1.00)** | | **0.55 (0.34,0.92)** | | **0.48 (0.28,0.83)** | |
| Atez | **2.13 (1.18,3.84)** | 1.08 (0.59,1.99) | | 1.12 (0.50,2.48) | | 1.03 (0.58,1.85) | | 1 | | 0.85 (0.50,1.43) | | 0.64 (0.41,1.00) | | 0.63 (0.36,1.10) | | 0.57 (0.33,1.01) | | **0.50 (0.27,0.91)** | |
| Ipi | **2.51 (1.56,4.03)** | 1.28 (0.78,2.10) | | 1.32 (0.65,2.69) | | 1.22 (0.77,1.93) | | 1.18 (0.70,1.99) | | 1 | | 0.76 (0.58,1.00) | | 0.74 (0.48,1.15) | | 0.68 (0.44,1.05) | | **0.58 (0.36,0.96)** | |
| Pla | **3.31 (2.25,4.87)** | **1.69 (1.12,2.55)** | | 1.74 (0.90,3.36) | | **1.61 (1.11,2.33)** | | 1.56 (1.00,2.43) | | 1.32 (1.00,1.74) | | 1 | | 0.98 (0.70,1.37) | | 0.89 (0.63,1.25) | | 0.77 (0.51,1.16) | |
| Durv | **3.39 (2.03,5.66)** | **1.73 (1.01,2.95)** | | 1.78 (0.85,3.73) | | **1.65 (1.00,2.72)** | | 1.59 (0.91,2.79) | | 1.35 (0.87,2.09) | | 1.02 (0.73,1.44) | | 1 | | 0.91 (0.56,1.48) | | 0.79 (0.46,1.34) | |
| Durv-Trem | **3.71 (2.22,6.21)** | **1.89 (1.11,3.23)** | | 1.95 (0.93,4.09) | | **1.80 (1.09,2.98)** | | 1.74 (0.99,3.06) | | 1.48 (0.95,2.29) | | 1.12 (0.80,1.57) | | 1.09 (0.68,1.77) | | 1 | | 0.86 (0.51,1.47) | |
| Atez-Tira | **4.30 (2.45,7.52)** | **2.19 (1.23,3.91)** | | **2.26 (1.04,4.89)** | | **2.09 (1.20,3.62)** | | **2.02 (1.10,3.69)** | | **1.71 (1.05,2.80)** | | 1.30 (0.86,1.95) | | 1.27 (0.75,2.15) | | 1.16 (0.68,1.97) | | 1 | |
| Matrix of pairwise comparisons of regimens on 7th months PFS (shown as hazard ratios and 95% confidence intervals). | | | | | | | | | | | | | | | | | | | |
|  | Serp | Nivo | | Pemb | | Adeb | | Atez | | Ipi | | Durv | | Durv-Trem | | Pla | | Atez-Tira | |
| SUCRA(%) | 88.32 | 72.32 | | 67.48 | | 61.70 | | 60.27 | | 38.10 | | 36.19 | | 33.10 | | 25.05 | | 17.48 | |
| Serp | 1 | 0.64 (0.27,1.48) | | 0.56 (0.30,1.03) | | **0.49 (0.28,0.86)** | | **0.48 (0.25,0.91)** | | **0.32 (0.19,0.52)** | | **0.30 (0.17,0.53)** | | **0.29 (0.17,0.50)** | | **0.27 (0.18,0.40)** | | **0.21 (0.12,0.37)** | |
| Nivo | 1.57 (0.68,3.64) | 1 | | 0.87 (0.36,2.10) | | 0.77 (0.33,1.78) | | 0.75 (0.31,1.83) | | 0.50 (0.22,1.10) | | 0.48 (0.21,1.09) | | 0.45 (0.20,1.04) | | **0.42 (0.20,0.87)** | | **0.33 (0.14,0.77)** | |
| Pemb | 1.79 (0.97,3.33) | 1.14 (0.48,2.75) | | 1 | | 0.88 (0.48,1.62) | | 0.86 (0.44,1.70) | | 0.57 (0.33,0.99) | | 0.55 (0.30,0.99) | | 0.52 (0.28,0.94) | | **0.48 (0.30,0.76)** | | **0.37 (0.20,0.70)** | |
| Adeb | **2.03 (1.16,3.56)** | 1.30 (0.56,2.99) | | 1.13 (0.62,2.08) | | 1 | | 0.97 (0.52,1.83) | | 0.64 (0.39,1.05) | | 0.62 (0.36,1.06) | | 0.58 (0.34,1.01) | | **0.54 (0.36,0.80)** | | **0.42 (0.24,0.75)** | |
| Atez | **2.08 (1.10,3.94)** | 1.33 (0.55,3.24) | | 1.16 (0.59,2.29) | | 1.03 (0.55,1.93) | | 1 | | 0.66 (0.37,1.17) | | 0.63 (0.34,1.18) | | 0.60 (0.32,1.12) | | **0.55 (0.34,0.91)** | | **0.43 (0.23,0.83)** | |
| Ipi | **3.17 (1.92,5.22)** | 2.02 (0.91,4.48) | | 1.76 (1.01,3.07) | | 1.56 (0.95,2.55) | | 1.52 (0.85,2.70) | | 1 | | 0.96 (0.60,1.55) | | 0.91 (0.56,1.48) | | 0.84 (0.62,1.13) | | 0.66 (0.39,1.11) | |
| Durv | **3.29 (1.90,5.72)** | 2.10 (0.92,4.82) | | 1.83 (1.01,3.34) | | 1.62 (0.94,2.79) | | 1.58 (0.85,2.94) | | 1.04 (0.64,1.68) | | 1 | | 0.95 (0.55,1.62) | | 0.87 (0.60,1.27) | | 0.69 (0.39,1.21) | |
| Durv-Trem | **3.47 (2.00,6.05)** | 2.22 (0.96,5.09) | | 1.94 (1.06,3.54) | | 1.71 (0.99,2.95) | | 1.67 (0.89,3.11) | | 1.10 (0.68,1.78) | | 1.06 (0.62,1.80) | | 1 | | 0.92 (0.63,1.35) | | 0.72 (0.41,1.28) | |
| Pla | **3.77 (2.52,5.63)** | **2.40 (1.15,5.03)** | | **2.10 (1.32,3.35)** | | **1.85 (1.25,2.74)** | | **1.81 (1.10,2.96)** | | 1.19 (0.88,1.60) | | 1.14 (0.78,1.67) | | 1.08 (0.74,1.59) | | 1 | | 0.78 (0.51,1.20) | |
| Atez-Tira | **4.80 (2.67,8.62)** | **3.06 (1.30,7.18)** | | **2.67 (1.42,5.03)** | | **2.36 (1.33,4.21)** | | **2.30 (1.20,4.41)** | | 1.52 (0.90,2.54) | | 1.46 (0.83,2.57) | | 1.38 (0.78,2.44) | | 1.27 (0.83,1.95) | | 1 | |
| Matrix of pairwise comparisons of regimens on 8th months PFS (shown as hazard ratios and 95% confidence intervals). | | | | | | | | | | | | | | | | | | | |
|  | Serp | Nivo | | Pemb | | Adeb | | Atez | | Durv | | Durv-Trem | | Ipi | | Pla | | Atez-Tira | |
| SUCRA(%) | 83.35 | 73.41 | | 66.80 | | 62.54 | | 56.77 | | 46.84 | | 41.15 | | 37.78 | | 20.69 | | 10.68 | |
| Serp | 1 | 0.77 (0.28,2.06) | | 0.63 (0.32,1.24) | | 0.58 (0.31,1.06) | | 0.51 (0.25,1.01) | | **0.41 (0.23,0.74)** | | **0.34 (0.19,0.62)** | | **0.34 (0.20,0.58)** | | **0.26 (0.17,0.39)** | | **0.16 (0.09,0.30)** | |
| Nivo | 1.31 (0.49,3.51) | 1 | | 0.83 (0.30,2.31) | | 0.75 (0.28,2.02) | | 0.66 (0.23,1.87) | | 0.54 (0.20,1.43) | | 0.45 (0.17,1.19) | | 0.44 (0.17,1.13) | | **0.33 (0.14,0.81)** | | **0.21 (0.08,0.57)** | |
| Pemb | 1.58 (0.81,3.10) | 1.21 (0.43,3.38) | | 1 | | 0.91 (0.46,1.78) | | 0.80 (0.38,1.68) | | 0.65 (0.34,1.25) | | 0.54 (0.28,1.04) | | 0.53 (0.29,0.97) | | **0.40 (0.24,0.67)** | | **0.25 (0.13,0.50)** | |
| Adeb | 1.74 (0.94,3.20) | 1.33 (0.50,3.57) | | 1.10 (0.56,2.15) | | 1 | | 0.88 (0.44,1.75) | | 0.72 (0.40,1.29) | | 0.59 (0.33,1.07) | | 0.58 (0.34,1.00) | | **0.44 (0.29,0.68)** | | **0.28 (0.15,0.52)** | |
| Atez | 1.98 (0.99,3.94) | 1.51 (0.54,4.27) | | 1.25 (0.59,2.63) | | 1.14 (0.57,2.26) | | 1 | | 0.81 (0.42,1.59) | | 0.67 (0.34,1.32) | | 0.66 (0.35,1.24) | | **0.50 (0.29,0.86)** | | **0.32 (0.16,0.64)** | |
| Durv | **2.43 (1.35,4.39)** | 1.86 (0.70,4.93) | | 1.54 (0.80,2.96) | | 1.40 (0.78,2.52) | | 1.23 (0.63,2.40) | | 1 | | 0.83 (0.47,1.47) | | 0.82 (0.49,1.37) | | **0.62 (0.42,0.93)** | | **0.39 (0.21,0.71)** | |
| Durv-Trem | **2.93 (1.61,5.33)** | 2.25 (0.84,5.98) | | 1.86 (0.96,3.60) | | 1.69 (0.93,3.06) | | 1.48 (0.76,2.92) | | 1.21 (0.68,2.15) | | 1 | | 0.98 (0.58,1.66) | | 0.75 (0.50,1.13) | | 0.47 (0.26,0.87) | |
| Ipi | **2.98 (1.73,5.13)** | 2.28 (0.89,5.88) | | 1.89 (1.03,3.47) | | 1.72 (1.00,2.94) | | 1.51 (0.81,2.82) | | 1.23 (0.73,2.06) | | 1.02 (0.60,1.72) | | 1 | | 0.76 (0.55,1.05) | | 0.48 (0.27,0.84) | |
| Pla | **3.92 (2.54,6.05)** | **3.00 (1.23,7.30)** | | **2.48 (1.48,4.16)** | | **2.26 (1.47,3.46)** | | **1.98 (1.16,3.39)** | | **1.61 (1.08,2.41)** | | 1.34 (0.89,2.02) | | 1.31 (0.95,1.82) | | 1 | | 0.63 (0.40,0.99) | |
| Atez-Tira | **6.22 (3.33,11.63)** | **4.76 (1.76,12.90)** | | **3.94 (1.98,7.82)** | | **3.58 (1.92,6.67)** | | **3.15 (1.56,6.35)** | | **2.56 (1.40,4.68)** | | **2.12 (1.15,3.91)** | | **2.09 (1.20,3.64)** | | **1.59 (1.01,2.49)** | | 1 | |
| Matrix of pairwise comparisons of regimens on 9th months PFS (shown as hazard ratios and 95% confidence intervals). | | | | | | | | | | | | | | | | | | | |
|  | Serp | Nivo | | Adeb | | Pemb | | Durv | | Durv-Trem | | Ipi | | Atez | | Pla | | Atez-Tira | |
| SUCRA(%) | 79.84 | 72.54 | | 68.49 | | 65.76 | | 48.33 | | 44.76 | | 43.65 | | 43.50 | | 17.26 | | 15.87 | |
| Serp | I | 0.85 (0.30,2.41) | | 0.75 (0.38,1.48) | | 0.71 (0.33,1.51) | | **0.46 (0.24,0.87)** | | **0.45 (0.24,0.85)** | | **0.45 (0.25,0.81)** | | **0.45 (0.21,0.92)** | | **0.28 (0.18,0.44)** | | **0.24 (0.12,0.46)** | |
| Nivo | 1.18 (0.41,3.33) | F | | 0.89 (0.31,2.56) | | 0.83 (0.27,2.54) | | 0.54 (0.19,1.52) | | 0.53 (0.19,1.49) | | 0.53 (0.19,1.44) | | 0.52 (0.17,1.57) | | **0.33 (0.13,0.84)** | | **0.28 (0.10,0.79)** | |
| Adeb | 1.33 (0.68,2.61) | 1.13 (0.39,3.26) | | A | | 0.94 (0.43,2.06) | | 0.61 (0.31,1.19) | | 0.59 (0.30,1.16) | | 0.59 (0.32,1.11) | | 0.59 (0.28,1.26) | | **0.37 (0.23,0.61)** | | **0.31 (0.16,0.62)** | |
| Pemb | 1.41 (0.66,3.00) | 1.20 (0.39,3.65) | | 1.06 (0.49,2.32) | | G | | 0.65 (0.30,1.37) | | 0.63 (0.30,1.34) | | 0.63 (0.31,1.28) | | 0.63 (0.27,1.44) | | **0.40 (0.22,0.72)** | | **0.33 (0.15,0.72)** | |
| Durv | **2.18 (1.15,4.14)** | 1.86 (0.66,5.25) | | 1.65 (0.84,3.21) | | 1.55 (0.73,3.28) | | C | | 0.98 (0.52,1.84) | | 0.98 (0.55,1.75) | | 0.97 (0.47,2.01) | | **0.61 (0.39,0.96)** | | **0.52 (0.27,0.99)** | |
| Durv-Trem | **2.23 (1.18,4.23)** | 1.90 (0.67,5.37) | | 1.68 (0.86,3.29) | | 1.58 (0.75,3.36) | | 1.02 (0.54,1.93) | | D | | 1.00 (0.56,1.79) | | 0.99 (0.48,2.05) | | **0.63 (0.40,0.98)** | | 0.53 (0.27,1.01) | |
| Ipi | **2.23 (1.24,4.03)** | 1.90 (0.69,5.21) | | 1.68 (0.90,3.14) | | 1.58 (0.78,3.22) | | 1.02 (0.57,1.83) | | 1.00 (0.56,1.79) | | E | | 0.99 (0.50,1.97) | | **0.63 (0.43,0.91)** | | **0.53 (0.29,0.97)** | |
| Atez | **2.25 (1.08,4.66)** | 1.91 (0.64,5.72) | | 1.69 (0.79,3.60) | | 1.59 (0.70,3.65) | | 1.03 (0.50,2.12) | | 1.01 (0.49,2.08) | | 1.01 (0.51,1.99) | | B | | 0.63 (0.36,1.12) | | 0.53 (0.25,1.11) | |
| Pla | **3.56 (2.26,5.61)** | **3.03 (1.19,7.72)** | | **2.68 (1.63,4.41)** | | **2.52 (1.38,4.61)** | | **1.63 (1.04,2.55)** | | **1.59 (1.02,2.49)** | | **1.59 (1.09,2.32)** | | 1.58 (0.90,2.80) | | H | | 0.84 (0.52,1.35) | |
| Atez-Tira | **4.24 (2.19,8.19)** | **3.60 (1.26,10.31)** | | **3.19 (1.60,6.35)** | | **3.01 (1.39,6.48)** | | **1.94 (1.01,3.73)** | | 1.90 (0.99,3.65) | | **1.90 (1.03,3.48)** | | 1.89 (0.90,3.96) | | 1.19 (0.74,1.92) | | J | |
| Matrix of pairwise comparisons of regimens on 10th months PFS (shown as hazard ratios and 95% confidence intervals). | | | | | | | | | | | | | | | | | | | |
|  | Nivo | Serp | | Adeb | | Pemb | | Atez | | Ipi | | Durv | | Durv-Trem | | Pla | | Atez-Tira | |
| SUCRA(%) | 82.69 | 72.70 | | 71.55 | | 62.45 | | 48.99 | | 46.39 | | 43.86 | | 40.74 | | 16.17 | | 14.47 | |
| Nivo | 1 | 0.69 (0.20,2.35) | | 0.67 (0.19,2.40) | | 0.53 (0.14,1.95) | | 0.39 (0.11,1.42) | | 0.35 (0.10,1.18) | | 0.35 (0.10,1.20) | | 0.32 (0.09,1.11) | | **0.20 (0.06,0.61)** | | **0.16 (0.05,0.56)** | |
| Serp | 1.46 (0.43,5.00) | 1 | | 0.97 (0.46,2.06) | | 0.77 (0.35,1.69) | | 0.56 (0.26,1.24) | | 0.51 (0.28,0.96) | | 0.51 (0.26,0.99) | | 0.47 (0.24,0.92) | | **0.28 (0.18,0.46)** | | **0.23 (0.12,0.46)** | |
| Adeb | 1.50 (0.42,5.39) | 1.03 (0.48,2.17) | | 1 | | 0.79 (0.33,1.87) | | 0.58 (0.25,1.37) | | 0.53 (0.26,1.07) | | 0.52 (0.24,1.11) | | 0.48 (0.23,1.03) | | **0.29 (0.16,0.52)** | | **0.24 (0.11,0.52)** | |
| Pemb | 1.89 (0.51,6.95) | 1.30 (0.59,2.84) | | 1.26 (0.53,2.99) | | 1 | | 0.73 (0.30,1.78) | | 0.66 (0.31,1.41) | | 0.66 (0.30,1.45) | | 0.61 (0.28,1.35) | | **0.37 (0.20,0.69)** | | **0.30 (0.14,0.68)** | |
| Atez | 2.58 (0.70,9.48) | 1.77 (0.81,3.87) | | 1.73 (0.73,4.07) | | 1.37 (0.56,3.33) | | 1 | | 0.91 (0.43,1.92) | | 0.90 (0.41,1.98) | | 0.84 (0.38,1.84) | | **0.50 (0.27,0.94)** | | **0.41 (0.19,0.92)** | |
| Ipi | 2.85 (0.85,9.55) | 1.95 (1.05,3.63) | | 1.90 (0.93,3.88) | | 1.51 (0.71,3.19) | | 1.10 (0.52,2.33) | | 1 | | 0.99 (0.53,1.86) | | 0.92 (0.49,1.73) | | **0.56 (0.37,0.84)** | | **0.46 (0.24,0.87)** | |
| Durv | 2.88 (0.84,9.89) | 1.97 (1.01,3.85) | | 1.92 (0.90,4.09) | | 1.52 (0.69,3.36) | | 1.11 (0.51,2.45) | | 1.01 (0.54,1.89) | | 1 | | 0.93 (0.47,1.83) | | **0.56 (0.35,0.90)** | | **0.46 (0.23,0.92)** | |
| Durv-Trem | 3.09 (0.90,10.66) | 2.12 (1.08,4.15) | | 2.07 (0.97,4.41) | | 1.64 (0.74,3.62) | | 1.20 (0.54,2.64) | | 1.09 (0.58,2.04) | | 1.08 (0.55,2.12) | | 1 | | **0.60 (0.37,0.98)** | | **0.50 (0.25,0.99)** | |
| Pla | **5.13 (1.64,16.02)** | **3.51 (2.20,5.62)** | | **3.42 (1.91,6.15)** | | **2.71 (1.44,5.10)** | | **1.98 (1.06,3.72)** | | **1.80 (1.20,2.71)** | | **1.78 (1.11,2.87)** | | **1.66 (1.02,2.68)** | | 1 | | 0.82 (0.50,1.36) | |
| Atez-Tira | **6.23 (1.80,21.61)** | **4.27 (2.15,8.47)** | | **4.16 (1.93,8.98)** | | **3.30 (1.47,7.37)** | | **2.41 (1.08,5.37)** | | **2.19 (1.15,4.17)** | | **2.17 (1.09,4.32)** | | **2.01 (1.01,4.03)** | | 1.22 (0.74,2.00) | | 1 | |
| Matrix of pairwise comparisons of regimens on 11th months PFS (shown as hazard ratios and 95% confidence intervals). | | | | | | | | | | | | | | | | | | | |
|  | Serp | Nivo | | Pemb | | Adeb | | Durv | | Durv-Trem | | Atez | | Ipi | | Pla | | Atez-Tira | |
| SUCRA(%) | 74.17 | 73.12 | | 67.65 | | 66.62 | | 56.39 | | 54.15 | | 46.91 | | 34.86 | | 14.50 | | 11.62 | |
| Serp | 1 | 1.00 (0.28,3.56) | | 0.85 (0.35,2.03) | | 0.83 (0.38,1.81) | | 0.66 (0.31,1.38) | | 0.62 (0.30,1.32) | | 0.52 (0.23,1.20) | | 0.36 (0.19,0.70) | | **0.25 (0.15,0.41)** | | **0.19 (0.09,0.40)** | |
| Nivo | 1.00 (0.28,3.54) | 1 | | 0.85 (0.22,3.29) | | 0.83 (0.23,3.03) | | 0.66 (0.18,2.36) | | 0.62 (0.17,2.24) | | 0.52 (0.14,1.98) | | 0.36 (0.11,1.25) | | **0.25 (0.08,0.79)** | | **0.19 (0.05,0.69)** | |
| Pemb | 1.18 (0.49,2.82) | 1.18 (0.30,4.60) | | 1 | | 0.98 (0.39,2.45) | | 0.77 (0.32,1.89) | | 0.74 (0.30,1.80) | | 0.62 (0.23,1.62) | | 0.43 (0.19,0.98) | | **0.29 (0.14,0.59)** | | **0.23 (0.09,0.55)** | |
| Adeb | 1.21 (0.55,2.62) | 1.21 (0.33,4.43) | | 1.02 (0.41,2.56) | | 1 | | 0.79 (0.36,1.76) | | 0.75 (0.34,1.68) | | 0.63 (0.26,1.52) | | 0.44 (0.21,0.90) | | **0.30 (0.17,0.54)** | | **0.23 (0.11,0.51)** | |
| Durv | 1.52 (0.72,3.20) | 1.53 (0.42,5.48) | | 1.29 (0.53,3.15) | | 1.26 (0.57,2.81) | | 1 | | 0.95 (0.44,2.05) | | 0.80 (0.34,1.86) | | 0.55 (0.28,1.10) | | **0.38 (0.22,0.65)** | | **0.29 (0.14,0.62)** | |
| Durv-Trem | 1.60 (0.76,3.38) | 1.60 (0.45,5.78) | | 1.36 (0.56,3.32) | | 1.33 (0.60,2.96) | | 1.05 (0.49,2.27) | | 1 | | 0.84 (0.36,1.97) | | 0.58 (0.29,1.16) | | **0.40 (0.23,0.69)** | | **0.31 (0.14,0.66)** | |
| Atez | 1.91 (0.83,4.39) | 1.92 (0.51,7.27) | | 1.62 (0.62,4.26) | | 1.59 (0.66,3.83) | | 1.26 (0.54,2.95) | | 1.19 (0.51,2.81) | | 1 | | 0.70 (0.32,1.52) | | **0.48 (0.25,0.92)** | | **0.37 (0.16,0.86)** | |
| Ipi | **2.74 (1.42,5.31)** | 2.75 (0.80,9.43) | | 2.33 (1.02,5.29) | | 2.28 (1.11,4.68) | | 1.80 (0.91,3.58) | | 1.71 (0.86,3.41) | | 1.43 (0.66,3.12) | | 1 | | 0.68 (0.45,1.04) | | 0.53 (0.27,1.04) | |
| Pla | **4.02 (2.42,6.69)** | **4.03 (1.26,12.84)** | | **3.41 (1.68,6.91)** | | **3.34 (1.85,6.00)** | | **2.64 (1.54,4.55)** | | **2.51 (1.46,4.33)** | | **2.10 (1.09,4.05)** | | 1.47 (0.96,2.23) | | 1 | | 0.78 (0.46,1.31) | |
| Atez-Tira | **5.19 (2.49,10.78)** | **5.20 (1.46,18.56)** | | **4.40 (1.82,10.61)** | | **4.30 (1.96,9.46)** | | **3.41 (1.60,7.25)** | | **3.24 (1.52,6.91)** | | **2.71 (1.17,6.29)** | | 1.89 (0.97,3.70) | | 1.29 (0.76,2.18) | | 1 | |
| Matrix of pairwise comparisons of regimens on 12th months PFS (shown as hazard ratios and 95% confidence intervals). | | | | | | | | | | | | | | | | | | | |
|  | Pemb | Durv | | Adeb | | Durv-Trem | | Nivo | | Serp | | Atez | | Ipi | | Pla | | Atez-Tira | |
| SUCRA(%) | 75.34 | 68.01 | | 67.20 | | 65.15 | | 59.64 | | 53.83 | | 49.85 | | 33.26 | | 14.85 | | 12.86 | |
| Pemb | 1 | 0.81 (0.28,2.31) | | 0.79 (0.28,2.27) | | 0.75 (0.26,2.14) | | 0.54 (0.14,2.15) | | 0.66 (0.24,1.76) | | 0.50 (0.16,1.54) | | **0.29 (0.11,0.76)** | | **0.20 (0.09,0.47)** | | **0.16 (0.06,0.44)** | |
| Durv | 1.23 (0.43,3.51) | 1 | | 0.98 (0.40,2.37) | | 0.92 (0.38,2.24) | | 0.67 (0.19,2.34) | | 0.81 (0.36,1.81) | | 0.62 (0.24,1.63) | | **0.36 (0.16,0.78)** | | **0.25 (0.14,0.47)** | | **0.20 (0.09,0.45)** | |
| Adeb | 1.26 (0.44,3.60) | 1.02 (0.42,2.47) | | 1 | | 0.94 (0.39,2.29) | | 0.68 (0.19,2.40) | | 0.82 (0.37,1.86) | | 0.63 (0.24,1.67) | | **0.36 (0.17,0.80)** | | **0.26 (0.14,0.48)** | | **0.20 (0.09,0.46)** | |
| Durv-Trem | 1.33 (0.47,3.81) | 1.08 (0.45,2.61) | | 1.06 (0.44,2.57) | | 1 | | 0.72 (0.20,2.54) | | 0.87 (0.39,1.96) | | 0.67 (0.25,1.77) | | **0.39 (0.18,0.85)** | | **0.27 (0.15,0.51)** | | **0.21 (0.09,0.49)** | |
| Nivo | 1.85 (0.47,7.36) | 1.50 (0.43,5.28) | | 1.47 (0.42,5.19) | | 1.39 (0.39,4.90) | | 1 | | 1.21 (0.36,4.06) | | 0.93 (0.25,3.49) | | 0.54 (0.16,1.77) | | 0.38 (0.13,1.13) | | 0.30 (0.09,1.01) | |
| Serp | 1.53 (0.57,4.09) | 1.24 (0.55,2.77) | | 1.21 (0.54,2.73) | | 1.14 (0.51,2.57) | | 0.82 (0.25,2.76) | | 1 | | 0.77 (0.31,1.89) | | 0.44 (0.22,0.89) | | 0.31 (0.19,0.52) | | 0.24 (0.12,0.52) | |
| Atez | 1.99 (0.65,6.09) | 1.61 (0.61,4.23) | | 1.58 (0.60,4.16) | | 1.49 (0.57,3.92) | | 1.07 (0.29,4.02) | | 1.30 (0.53,3.20) | | 1 | | 0.57 (0.24,1.38) | | 0.41 (0.19,0.85) | | 0.32 (0.13,0.80) | |
| Ipi | **3.46 (1.31,9.10)** | **2.80 (1.28,6.14)** | | **2.75 (1.25,6.05)** | | **2.59 (1.18,5.70)** | | 1.87 (0.57,6.16) | | 2.27 (1.12,4.57) | | 1.74 (0.72,4.19) | | 1 | | 0.71 (0.44,1.14) | | 0.55 (0.27,1.15) | |
| Pla | **4.90 (2.11,11.38)** | **3.97 (2.13,7.40)** | | **3.89 (2.07,7.31)** | | **3.68 (1.97,6.87)** | | 2.65 (0.89,7.90) | | 3.21 (1.92,5.37) | | 2.47 (1.18,5.16) | | 1.42 (0.88,2.28) | | 1 | | 0.78 (0.45,1.36) | |
| Atez-Tira | **6.25 (2.29,17.07)** | **5.06 (2.21,11.61)** | | **4.96 (2.15,11.43)** | | **4.68 (2.04,10.77)** | | 3.37 (0.99,11.47) | | 4.09 (1.93,8.68) | | 3.14 (1.25,7.88) | | 1.81 (0.87,3.74) | | 1.27 (0.74,2.21) | | 1 | |

Abbreviations: Nivo:nivolumab;Atez-Tira:atezolizumab+tiragolumab;Atez:atezolizumab;Serp:serplulimab;Durv:Durvalumab;Durv-Trem:Durvalumab+tremelimumab; Pla:Placebo;Adeb:Adebrelimab; Pemb:Pembrolizumab; Ipi:ipilimumab.

Table S8. Baseline Clinical and disease Characteristics of Trials Included in the Network Meta-analysis.

|  |  | **CA184-156(n=954)** | | **IMpower133(n=403)** | | | **KEYNOTE-604(n=446)** | |  |
| --- | --- | --- | --- | --- | --- | --- | --- | --- | --- |
|  |  | Ipi Group  (n = 478) | Pla Group  (n = 476) | Atez Group  (n=201) | | Pla Group  (n=202) | Pemb Group  (n=223) | Pla Group  (n=223) |  |
| Age | | | | | | | | | |
|  | <65 | 63% | 58% | 55% | | 53% | 50% | 45% |  |
|  | ≥65 | 37% | 42% | 45% | | 48% | 50% | 55% |  |
| Gender | | | | | | | | | |
|  | Male | 66% | 68% | 64% | | 65% | 67% | 63% |  |
|  | Female | 34% | 32% | 36% | | 35% | 33% | 37% |  |
| Smoking status | | | | | | | | | |
|  | Current or former | 56% | 57% | 96% | | 99% | 97% | 96% |  |
|  | Never or unknow | 44% | 43% | 5% | | 2% | 4% | 4% |  |
| ECOG performance-status score | | | | | | | | | |
|  | 0% | 29% | 31% | 36% | | 33% | 26% | 25% |  |
|  | ≥1 | 71% | 69% | 64% | | 67% | 74% | 75% |  |
| Brain metastases at enrollment | | | | | | | | | |
|  | Yes | 12% | 10% | 9% | | 9% | 15% | 10% |  |
|  | No | 88% | 90% | 92% | | 91% | 86% | 90% |  |
| Liver metastases at enrollment | | | | |  | | | | |
|  | Yes | NR | NR | NR | | NR | 42% | 41% |  |
|  | No | NR | NR | NR | | NR | 58% | 59% |  |
|  |  |  |  |  | |  |  |  |  |
|  |  | **CAPSTONE-1(n=462)** | | **ASTRUM-005(n=585)** | | | **CASPIAN(n=805)** | | |
|  |  | Adeb Group  (n=230) | Pla Group  (n=232) | Serp Group  (n = 389) | | Pla Group  (n = 196) | Durv-Trem Group  (n=268) | Durv Group  (n=268) | Pla Group  (n=269) |
| Age | | | | | | | | | |
|  | <65 | 67% | 63% | 60% | | 61% | 57% | 62% | 58% |
|  | ≥65 | 33% | 37% | 40% | | 39% | 43% | 38% | 42% |
| Gender | | | | | | | | | |
|  | Male | 80% | 81% | 82% | | 84% | 75% | 71% | 68% |
|  | Female | 20% | 19% | 19% | | 16% | 25% | 29% | 32% |
| Smoking status | | | | | | | | | |
|  | Current or former | 78% | 13% | 79% | | 82% | 94% | 92% | 94% |
|  | Never or unknow | 22% | 87% | 21% | | 18% | 6% | 8% | 6% |
| ECOG performance-status score | | | | | | | | | |
|  | 0% | 14% | 13% | 18% | | 16% | 41% | 37% | 33% |
|  | ≥1 | 86% | 87% | 82% | | 84% | 59% | 63% | 67% |
| Brain metastases at enrollment | | | | | | | | | |
|  | Yes | 2% | 2% | 13% | | 14% | 14% | 10% | 10% |
|  | No | 98% | 98% | 87% | | 86% | 86% | 90% | 90% |
| Liver metastases at enrollment | | | | | | | | | |
|  | Yes | 32% | 32% | 25% | | 26% | 44% | 40% | 39% |
|  | No | 68% | 68% | 75% | | 74% | 56% | 60% | 61% |
|  |  |  |  |  | |  |  |  |  |
|  |  | **EA5161(n=160)** | | **SKYSCRAPER-02(n=490)** | | | **CA184-041(n=130)** | | |
|  |  | Nivo Group  (n=80) | Pla Group  (n=80) | Atez-Tira Group  (n=243) | | Pla Group  (n=249) | Concurrent-Ipi Group  (n=45) | Phased-Ipi Group  (n=43) | Pla Group  (n=42) |
| Age | | | | | | | | | |
|  | <65 | NR | NR | 48% | | 47% | 81% | 69% | 80% |
|  | ≥65 | NR | NR | 52% | | 53% | 19% | 31% | 20% |
| Gender | | | | | | | | | |
|  | Male | 44% | 45% | 67% | | 66% | 77% | 76% | 73% |
|  | Female | 56% | 55% | 33% | | 34% | 23% | 24% | 27% |
| Smoking status | | | | | | | | | |
|  | Current or former | NR | NR | 96% | | 96% | 88% | 88% | 90% |
|  | Never or unknow | NR | NR | 4% | | 4% | 12% | 12% | 10% |
| ECOG performance-status score | | | | | | | | | |
|  | 0% | 49% | 51% | 35% | | 33% | 19% | 26% | 27% |
|  | ≥1 | 51% | 49% | 64% | | 67% | 81% | 74% | 73% |
| Brain metastases at enrollment | | | | | | | | | |
|  | Yes | NR | NR | 19% | | 19% | NR | NR | NR |
|  | No | NR | NR | 81% | | 81% | NR | NR | NR |
| Liver metastases at enrollment | | | | | | | | | |
|  | Yes | NR | NR | 37% | | 38% | NR | NR | NR |
|  | No | NR | NR | 63% | | 62% | NR | NR | NR |

Abbreviations: Nivo:nivolumab;Atez-Tira:atezolizumab+Atez-Tiragolumab;Atez:atezolizumab;Serp:serplulimab;Durv:Durvalumab;Durv-Trem:Durvalumab+tremelimumab; Pla:Placebo;Adeb:Adebrelimab; Pemb:Pembrolizumab; Ipi:ipilimumab; NR, not reporte.

Figure S1. Results of risk of bias assessment.

**A** History for overall survival


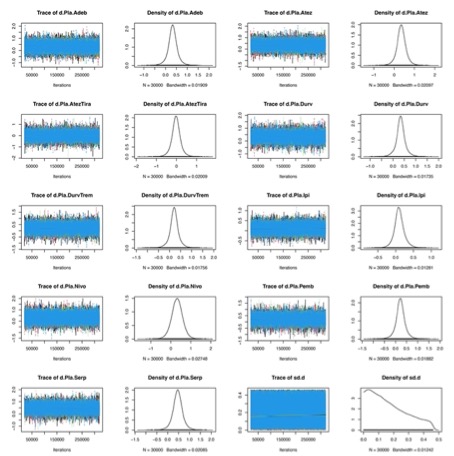


**B** History for progression-free survival


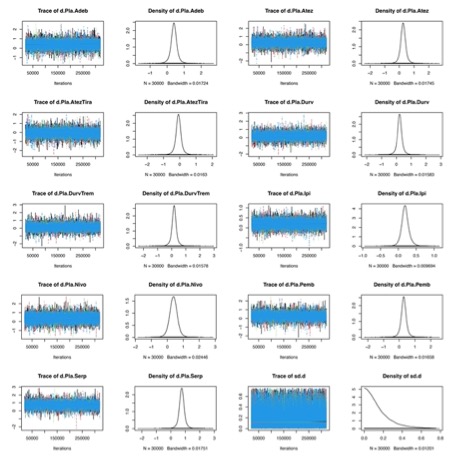


**C** History for objective response rate


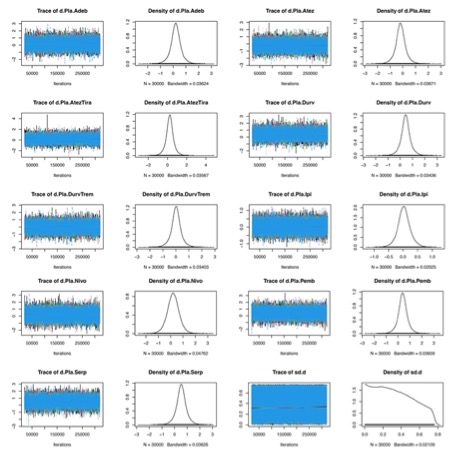


**D** History for grade ≥ 3 adverse events


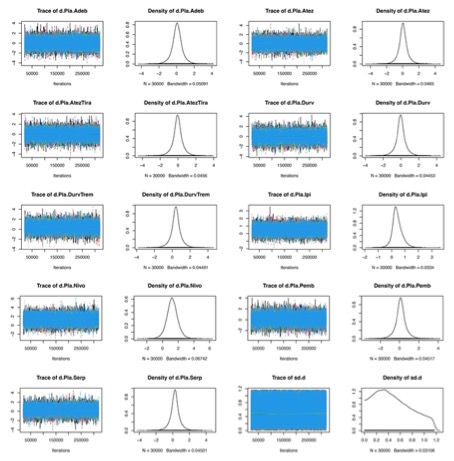


Figure S2. Convergence of the three Markov Chain Monte Carlo (MCMC) chains established by of the history feature for overall survival (A), progression-free survival (B), objective response rate (C), and grade ≥3 AEs (D).Abbreviations: Nivo: Nivolumab; Atez-Tira: Atezolizumab+Tiragolumab;Atez: Atezolizumab;Serp: Serplulimab;Durv: Durvalumab;Durv-Trem: Durvalumab+Tremelimumab;Pla: Placebo;Adeb: Adebrelimab;Pemb: Pembrolizumab;Ipi: Ipilimumab.

**A** Brooks-Gelman-Rubin diagnostic for overall survival


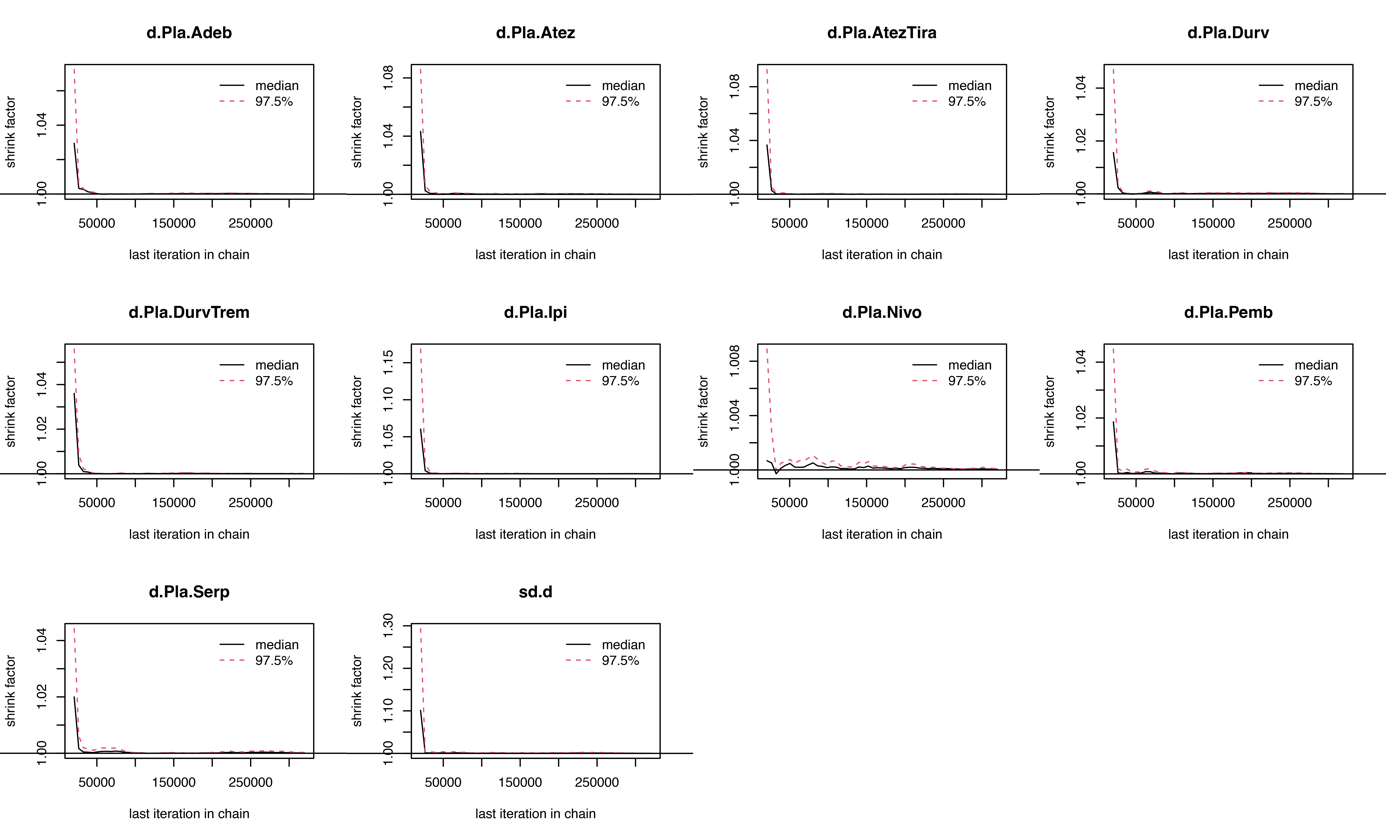


**B** Brooks-Gelman-Rubin diagnostic for progression-free survival

**
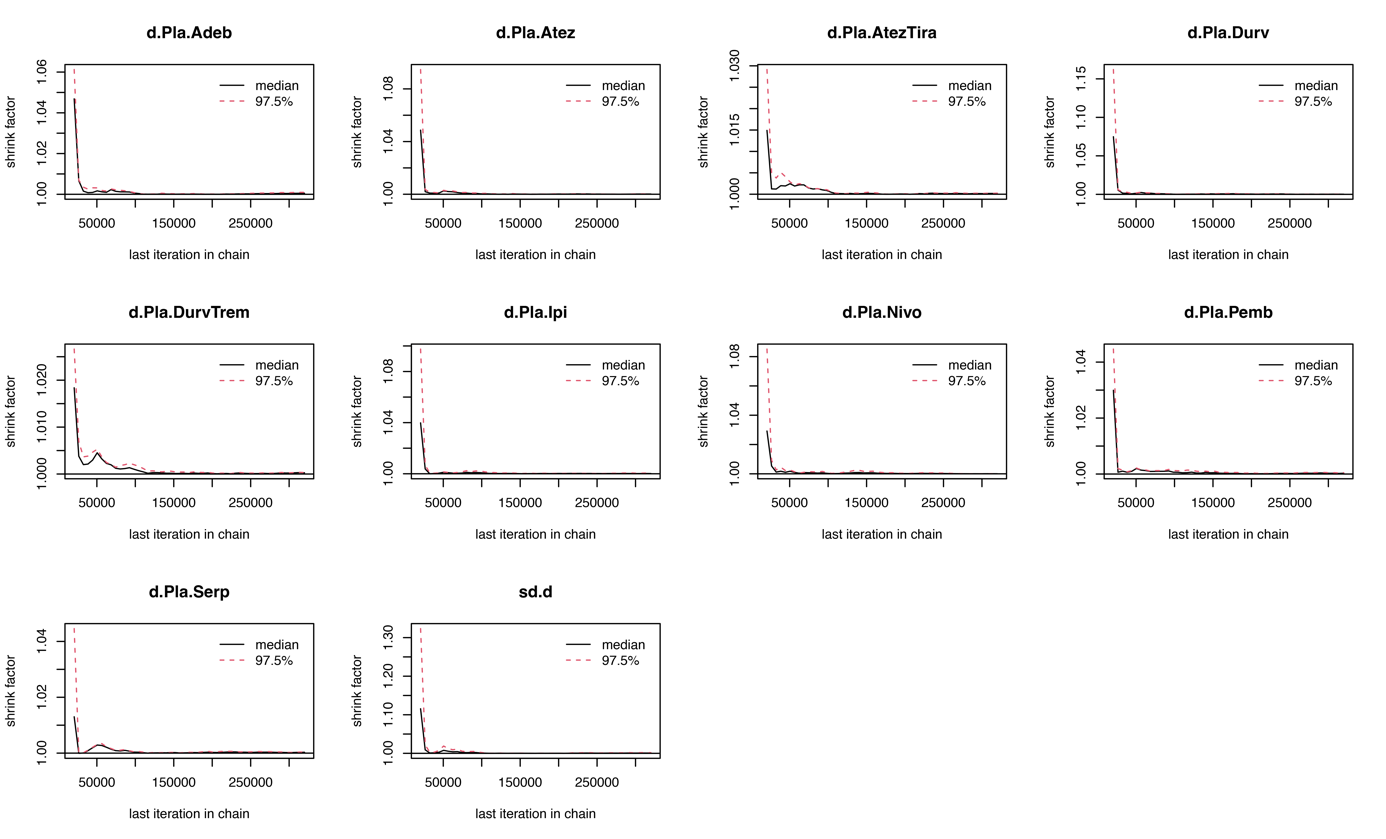
**

**C** Brooks-Gelman-Rubin diagnostic for objective response rate


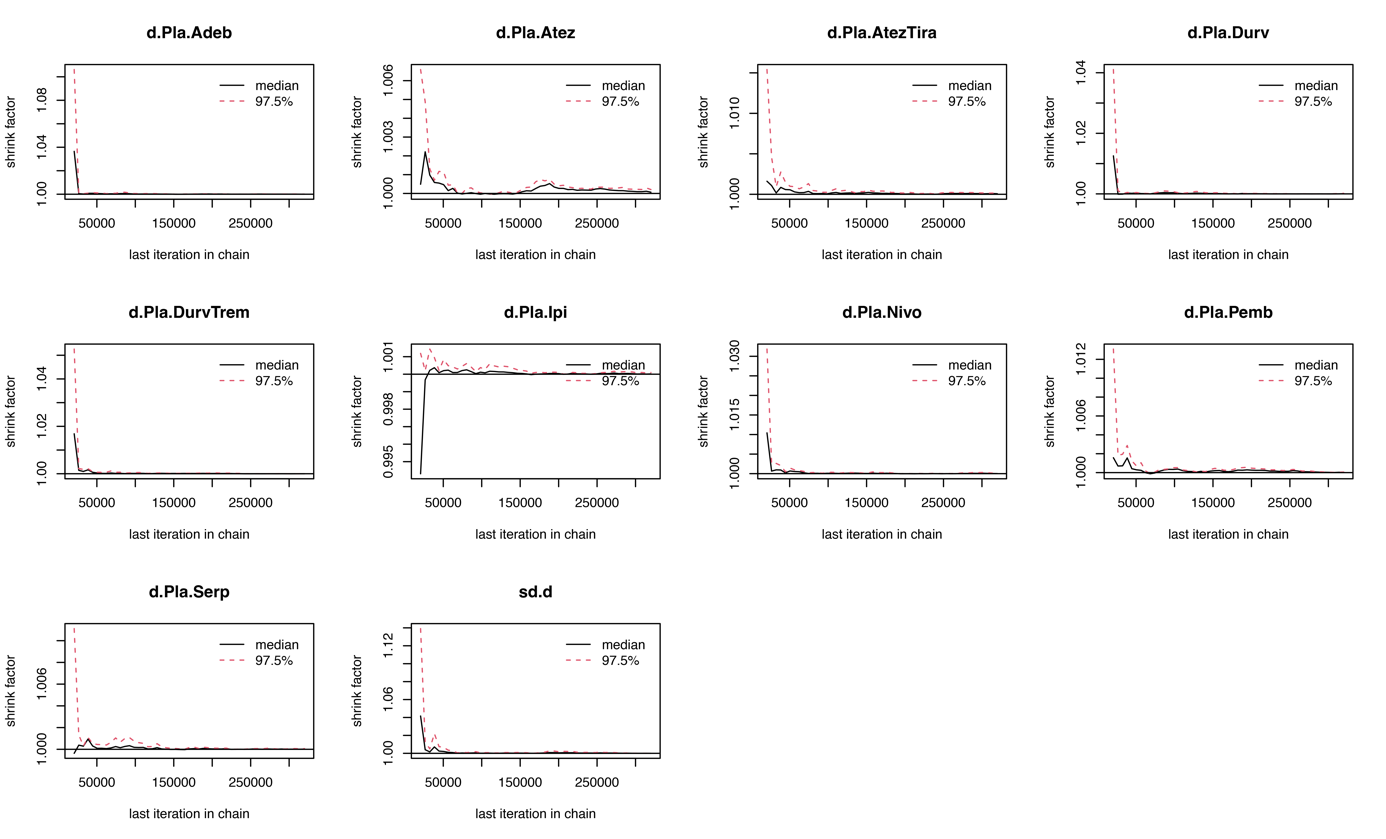


**D** Brooks-Gelman-Rubin diagnostic for grade ≥3 adverse events


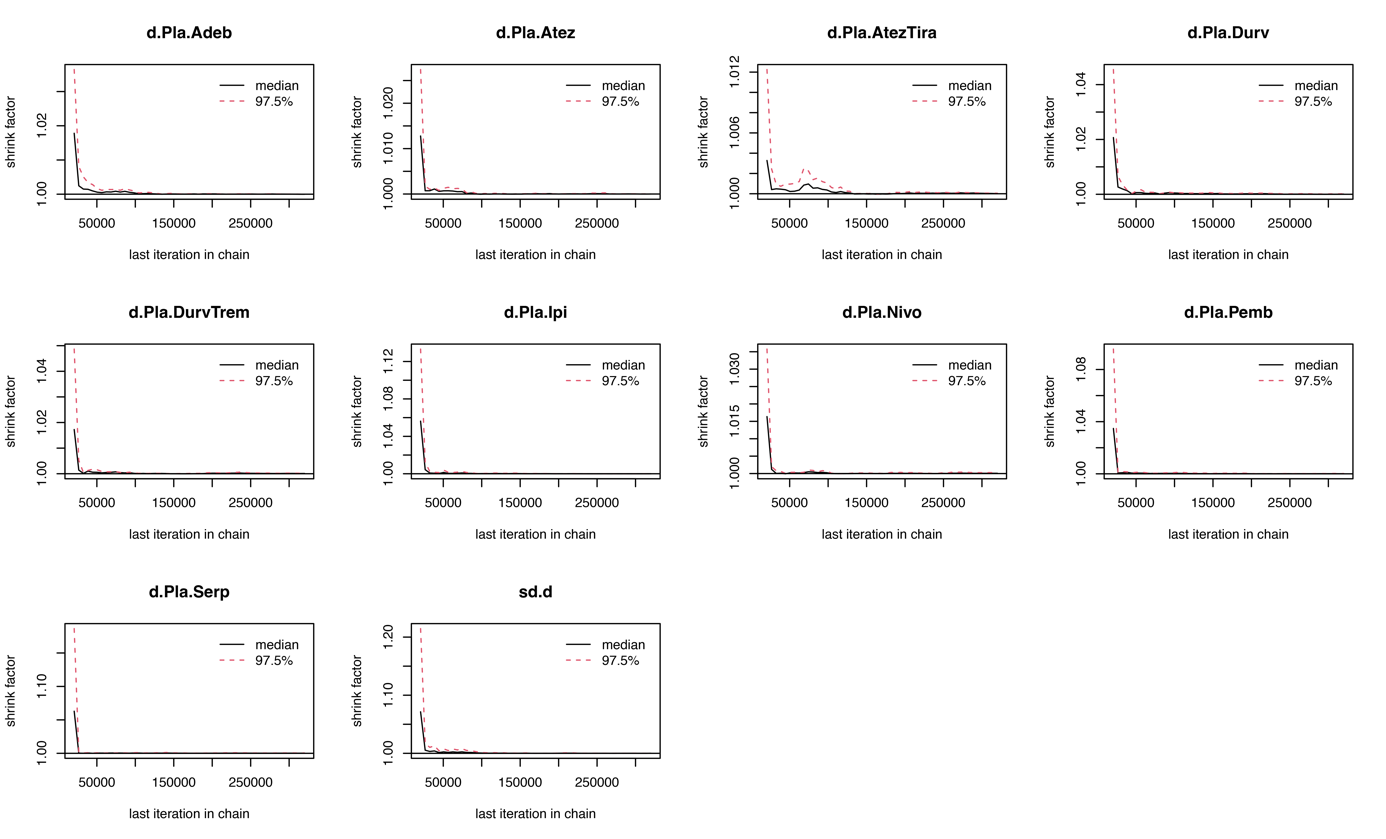
Figure S3. Convergence of the three Markov Chain Monte Carlo (MCMC) chains established by of the Brooks-Gelman-Rubin diagnostic for overall survival (A), progression-free survival (B), objective response rate (C), and grade ≥3 AEs (D). Abbreviations: Nivo: Nivolumab; Atez-Tira: Atezolizumab+Tiragolumab;Atez: Atezolizumab;Serp: Serplulimab;Durv: Durvalumab;Durv-Trem: Durvalumab+Tremelimumab;Pla: Placebo;Adeb: Adebrelimab;Pemb: Pembrolizumab;Ipi: Ipilimumab.


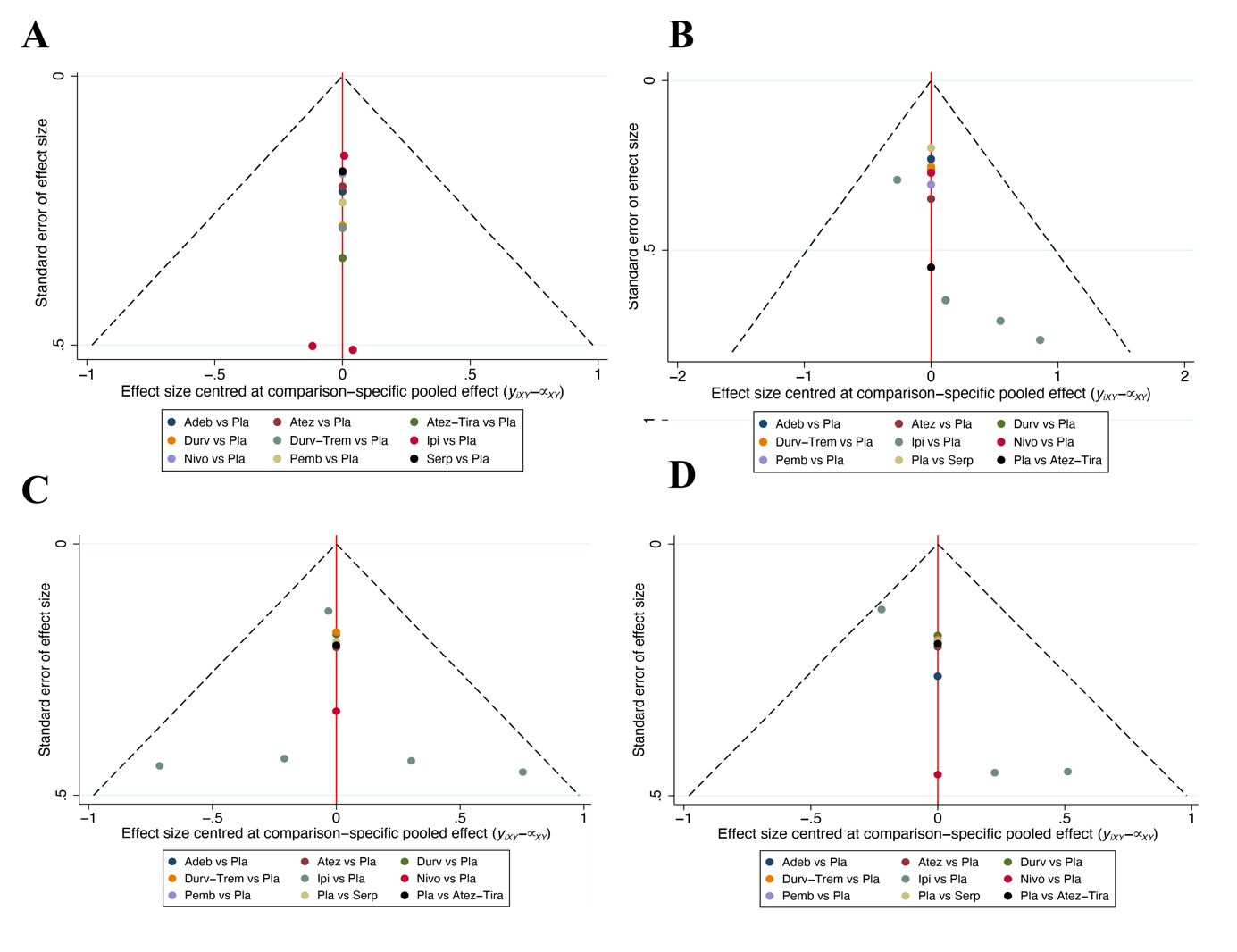


Figure S4. Funnel plot for the detection of small sample effect. (A) The funnel plot of OS;(B) The funnel plot of PFS; (C) The funnel plot of ORR; (D) The funnel plot of grade ≥3 AEs. Abbreviations: Nivo: Nivolumab; Atez-Tira: Atezolizumab+Tiragolumab; Atez: Atezolizumab; Serp: Serplulimab; Durv: Durvalumab; Durv-Trem: Durvalumab+Tremelimumab; Pla: Placebo; Adeb: Adebrelimab; Pemb: Pembrolizumab; Ipi: Ipilimumab.
